# Adrenarche, social cognition, and the development and evolution of autism spectrum traits

**DOI:** 10.3389/fpsyt.2025.1576392

**Published:** 2025-09-09

**Authors:** Cory Szakal, Bernard Crespi

**Affiliations:** Department of Biological Sciences, Simon Fraser University, Burnaby, BC, Canada

**Keywords:** adrenarche, autism, DHEA, DHEAS, middle childhood, evolutionary, juvenile stage

## Abstract

Autism Spectrum Disorder (ASD) is a heterogeneous neurodevelopmental condition characterized by underdeveloped social cognition, along with restricted interests and repetitive behaviors. ASD manifests through a range of genetic, environmental, and psychosocial factors, which influence brain development and lead to maladaptive social and behavioral processes. While early diagnosis is common, ASD traits can develop and express themselves through various stages of childhood, driven by dynamic changes in cognitive and social abilities in relation to stressors and challenges. A recent study reports genomic and psychological evidence for two different age-related trajectories of autism development, one early, and one later and near the time of adrenarche and middle childhood, around ages 7 - 10. Given that middle childhood represents a key period for the development of social cognition including complex theory of mind and peer relationships, that adrenarche mediates the origin and social adaptations of middle childhood, and that social challenges increase with its onset, we hypothesize that autism onset, expression and diagnoses during this period may involve alterations to adrenarche, and to its endocrinological and neurological bases. Adrenarche involves onset and increase in secretion of the androgens dehydroepiandrosterone (DHEA) and its sulfate (DHEAS). A series of systematic reviews was conducted to evaluate the hypothesis that DHEA or DHEAS levels are associated with ASD, autism spectrum traits, or aspects of brain development relevant to autism. The reviews showed that: (1) higher DHEA demonstrated evidence of positive associations with aspects of internalizing and externalizing, including social anxiety, with especially notable effects in girls, and (2) higher DHEA showed evidence of association with ASD diagnoses overall, as also indicated by a recent meta-analysis. These findings provide initial support for the hypothesis that alterations to the social adaptations associated with adrenarche, and DHEA levels in middle childhood may underlie a subtype of autism with diagnosis during this developmental period.

## 1 Introduction

Autism Spectrum Disorder (ASD) is a neurodevelopmental condition characterized by the underdevelopment of social cognition and the presence of restricted interests and repetitive behaviors (1). There is high variability in how individuals express ASD traits and in the severity with which those traits impact their lives, ranging from moderate effects on social interaction to severe social difficulties, and from simple repetitive behaviors to elaborate rituals or stereotypies (2). Such variation in autism trait expression may be related to the many genetic, environmental, and psychosocial factors that underlie its causes. ASD has a strong genetic component, with heritability estimated to be 70 - 80% (3). Over 800 common risk-related genes have been identified, each contributing small effects, indicating the presence of many alleles with minor influence that collectively add to the genetic aspect of the disorder (in addition to larger-affect mutations such as copy number variants) (4). In addition to genetic factors, environmental influences, including advanced parental age, maternal medication use, premature or difficult births, and exposure to toxins have also been indicated as causes of ASD (5). Psychosocial factors, including maternal education levels, parental immigration, maternal trauma and depression, invalidation, less responsive parenting, and poor home environments have also been linked to the emergence of ASD traits (6, 7).

Genetic, environmental, and psychosocial factors interact to influence the diverse onset and presentation of ASD traits (4–8). The wide diagnostic age range for autism, from infancy through adolescence and adulthood (9), suggests that alterations in different social and cognitive adaptations, at different developmental stages, may contribute to variability in symptom presentation and diagnosis timing. Children with more severe symptoms are often diagnosed earlier due to higher levels of parental concern about symptoms, with diagnostic delays also influenced by socioeconomic status; for example, children from rural areas or low-income families receive diagnoses significantly later than their urban or higher-income peers, often due to reduced access to specialists and limited parental awareness of early ASD indicators (10). Diagnostic timing also differs by gender, with females generally diagnosed later than males (11), likely due to the higher prevalence of camouflaging strategies, whereby autistic traits are unconsciously or consciously masked by mimicking social norms, rehearsing scripts, or suppressing atypical behaviors. Whereas a clinical diagnosis according to the fifth edition of the Diagnostic and Statistical Manual of Mental Disorders (DSM - 5) requires symptoms to be present in early childhood, they may not become obvious until social challenges exceed one’s capacities for dealing with them.

Variation among individuals in the severity, expression, and diagnostic age of autism reflect its diversity of genetic, endocrinological, neurological and psychological causes. The cause of autism in any given individual is, in turn, expected to reflect alterations to specific cognitive, affective and behavioral adaptations, in the context of the timing of specific developmental stages such as those that subserve social attention or the acquisition of language (12–14). In this adaptive-developmental context, some foundational social adaptations acquired in the pre-adult period, including complex theory of mind and the social reciprocities of peer friendships, develop predominantly during middle childhood, between the ages of about 7 and 10 (15–17). Indeed, in a longitudinal study, Osterhaus and Koerber (18) found that advanced theory of mind develops non-linearly in children, with a conceptual ‘milestone’ usually reached around age 7. Middle childhood thus appears to represent a critical period in social development (16, 17, 19), raising the question of whether alterations to the adaptive onset and completion of this stage, in the context of its higher level of social challenges, can represent important causes of variation in the symptoms of autism, especially with regard to later-diagnosed forms of this condition.

Consistent with the idea that middle childhood may represent an important ‘critical period’ for the development, expression, and diagnosis of autism, Zhang et al. (20) analyzed longitudinally collected data from four autism birth cohort studies and reported genomic and psychological evidence for two different genetically-based trajectories in the expression and diagnosis of autism, one relatively early, and the other overlapping broadly with middle childhood. These findings suggest that some notable proportion of autism cases may derive from genetically based alterations to this developmental period, which is initiated by adrenarche, the ‘awakening’ of the adrenal grands via the onset of secretion of the androgens DHEA and DHEAs.

In this article, we develop and evaluate the hypothesis that ASD and sets of ASD-related traits commonly arise from alterations to the timing of adrenarche and levels of DHEA, which affect social development during middle childhood. To evaluate this hypothesis, we address the following three questions:

1. What social adaptations develop during middle childhood in boys and girls, and how are they influenced by adrenarche timing and DHEA levels? Addressing this question will help to characterize the social adaptations that typify the human juvenile (middle childhood) stage.
2. Do alterations in adrenarche timing, and relatively high or low DHEA levels contribute to the manifestation of ASD traits by affecting the social-developmental adaptations that characterize this stage? Addressing this question will help in testing the hypothesis that some proportion of cases of ASD involve alterations to adrenarche, DHEA, DHEAS, and social development during this stage.
3. Are there links between ASD diagnoses, or ASD traits including repetitive behaviors and restricted interests, with altered adrenarche timing, leading to alterations in ASD phenotypes during middle childhood?

We first provide brief overviews of the adaptive, evolved stages of human social development, with a focus on adrenarche and its endocrinological basis in DHEA. Second, we systematically review studies examining social development in relation to DHEA and DHEAS (hence, DHEA(S)). DHEA(S) levels during adrenarche, to identify evidence salient to social-developmental adaptations influenced by variation in this hormone. This overview focuses on key social skills and behaviors that emerge in typically developing (TD) children and how variations in DHEA(S) levels may affect them. Next, we systematically review the literature on the relationship between alterations in adrenarche timing, DHEA(S) levels, and the manifestation of ASD. These reviews are intended to elucidate if and how hormonal factors may contribute to variation in social-developmental adaptations during middle childhood, potentially linking it to the emergence and expression of ASD in this period. Finally, we also review potential roles of adrenarche and DHEA(S) in repetitive behaviors and restricted interests, examining how these ASD-related traits may be impacted by mechanisms associated with DHEA(S).

### 1.1 Adaptive stage-specific human development

Early human development can be divided into four distinct stages: infancy, childhood, juvenility, and adolescence, each characterized by the acquisition of important social-developmental adaptations (12). Early literature on autism, particularly Kanner’s (21) seminal work, described autism as a condition evident from early infancy. Many studies have thus focused on infancy as the primary stage where alterations leading to ASD diagnoses manifest themselves. For example, Baron-Cohen’s (22) work focuses on prenatal events, in particular elevated prenatal testosterone levels, which show evidence of leading to the exaggeration of typical male traits, including systemizing abilities and attention to detail, with a reduced emphasis on typical female traits, including empathy and social communication. Considering that empathizing and systemizing are cognitive traits distributed continuously across the general population, they can be conceptualized as existing along a continuum, with extremes at both ends (23). However, these traits are not fixed in infancy, as they continue to undergo significant developmental changes throughout childhood and adolescence.

During infancy and early childhood, parental relationships are vital for survival, and thus maintaining caregiver attachment significantly shapes the acquisition of cognition and social skills. Attachment bonds foster a sense of safety, with related behaviors evolving from crying and clinging in infancy to using caregivers as a secure base for exploration (24). Through engagement and imitation, caregiver interactions foster emotional regulation, emotional reciprocity, and early language acquisition. Joint attention, through shared focus on objects or events via pointing, showing, or pretend play, establishes common reference points for communication and helps link words to meanings, facilitating early language development; problems with joint attention are common in children who develop ASD, characterized by reduced initiation, response, and difficulty connecting gaze to mental states (25). Impairments in language acquisition and social communication in ASD often become evident between 12 and 24 months, and contribute to diagnoses (26). Environmental influences, particularly from parenting, may also contribute to the acquisition of social adaptations, resulting in maladaptive behaviors that reflect ASD traits. For instance, childhood neglect and inconsistent parenting have been related to language delays (27), and ASD children whose parents demonstrate secure attachment show greater abilities in reciprocal communication and social problem-solving (24). These early relational dynamics may shape the timing and development of cognitive and social skills.

Similarly to language delays and social impairments, repetitive behaviors begin to manifest around 12 and 24 months and continue to develop throughout childhood (28). The developmental heterochrony hypothesis of ASD (12) posits that alterations in the timing and rate of development in ASD may lead to the prolonged expression of traits typically seen in younger individuals. For instance, restricted and repetitive behaviors, such as a preference for sameness and ritualistic actions, are common in typically developing children around 2–3 years of age but generally decline by age 11, whereas these traits persist in children with ASD. Given their prevalence in the general population, restricted interests and repetitive behaviors may serve adaptive functions in early childhood, such as helping to manage anxiety during significant developmental transitions; the persistence of these traits in ASD would thus reflect deviations from typical adaptive cognitive progression through developmental stages that potentially lead to maladaptive outcomes.

### 1.2 Adrenarche and social development during middle childhood

The onset of the middle childhood (sometimes called ‘juvenile’) stage, usually between six and eight years of age, coincides with significant transitions and enhancements in traits related to social capabilities, personality development, and sexual identity and behavior (15). As children enter school age around this time, they are given greater responsibilities in familial roles and develop increased social independence, as well as forming their first notable relationships with peers (29).

As with caregiver interactions during infancy and early childhood, peer relationships influence social learning and the development of adaptive capacities during middle childhood. Motivated by the exploration of new friendships and peer groups, children often face novel social challenges, including social rejection and peer victimization, which heightens risks for depression and social anxiety by reinforcing self-devaluation and social-evaluative threat (30). Such experiences can be highly aversive, and altered neural processing of social signals, such as reduced sensitivity to indifference or rejection, may be adaptive for facilitating social exploration (31). Additionally, successfully navigating complex social environments requires understanding and predicting the actions of others and making inferences about their thoughts, beliefs, and intentions, as facilitated by theory of mind (ToM). Enhanced ToM development occurs around age seven and allows for increasingly sophisticated social reasoning, which continues to develop through late elementary school with advancements in grasping ambiguity and social norms (18). Peer relationships during this period offer children essential opportunities to practice and refine social skills and ToM abilities; however, the absence of supportive peer relationships or experiences of social conflict can result in social withdrawal and hinder this developmental process (32, 33). Physiological and neurological changes in middle childhood may thus interact with social experiences to promote enduring patterns of social cognition and behavior.

Adrenarche defines the beginning of middle childhood, when the zona reticularis of the adrenal gland matures and begins to secrete substantially higher levels of androgens, particularly dehydroepiandrosterone (DHEA) and its sulfate (DHEAS) (34). Unique to humans and great apes, adrenarche apparently evolved in the context of extended childhood social-brain maturation via an extended period of social enculturation prior to sexual maturity (17). Social-developmental adaptations emerging during middle childhood include aspects of cognition (e.g. increased problem-solving, reasoning, mentalizing skills, self-regulation, executive functions such as inhibition and attention), motivation, and social behavior (15, 35). In terms of brain development, ongoing myelination and synaptic pruning refine neural connections during this period, resulting in faster communication between brain hemispheres and more targeted activation of specific brain regions and networks (19). These cognitive and neurological changes occur alongside somatic transformations including molar eruption, increased adiposity, and pronounced sex differences in body composition and vocal characteristics (36). Together, these cognitive, neurological, and somatic changes set the stage for new social and emotional challenges, positioning adrenarche as a critical period for developing and refining social adaptation mechanisms.

### 1.3 DHEA and its role in social adaptations

Dehydroepiandrosterone (DHEA) and its sulfate (DHEAS), key adrenarchal androgens, play multifaceted roles in supporting the development of cognitive processes during middle childhood, wherein the dramatic spike of levels defines adrenarche. As neurosteroids, DHEA and DHEAS can bind directly to neurotransmitter receptors, and have been found to modulate neural activity by acting as noncompetitive antagonists at GABAA receptors, reducing inhibitory neurotransmission, and as positive allosteric modulators at NMDA receptors, enhancing excitatory signaling, and facilitating dendritic growth, axonal elongation, and synaptic plasticity in response to environmental stimuli (16, 37). DHEA(S) administration in adults has been shown to reduce amygdala and hippocampus activity while enhancing their regulatory connectivity, leading to decreased emotional reactivity and altered memory for emotional stimuli; given the amygdala’s role in emotional salience and the hippocampus’s role in contextual memory and fear conditioning, DHEA(S) may thus help modulate negative responses to aversive emotional stimuli (38). Additionally, DHEA(S) functions as a stress hormone, with levels rising along with cortisol in response to stressful social situations, both being released by the hypothalamic-pituitary-adrenal (HPA) axis, underscoring its role in helping children adapt to social challenges (48). The anti-glucocorticoid properties of DHEA(S) counteract the neurotoxic effects of cortisol by reducing its impact on neural receptors, enhancing neuronal survival and protecting the hippocampus from stress-induced damage (37). Given the increased production of DHEA(S) during middle childhood, adrenarche is thought to modulate brain function in response to these new social demands, and variations in levels of these neurosteroids may influence neural and biochemical pathways that shape social integration (16, 17).

Early exposure to DHEA(S) through premature adrenarche (PA), defined as the early appearance of adrenarche-mediated somatic changes before about eight years of age in girls and nine years of age in boys (36), introduces shifts in the expected developmental timeline of hormonal changes, potentially impacting the acquisition of social and cognitive skills during middle childhood. While earlier pubertal timing supports earlier reproduction, the early maturation hypothesis suggests that PA may disadvantage children by limiting opportunities for gradual, adaptively staged socio-emotional development (48). PA has been linked to heightened depression, anxiety, and both internalizing and externalizing symptoms, with neuroimaging studies associating elevated DHEA(S) with neural markers of emotional dysregulation (34, 65). These early hormonal shifts can activate stress-sensitive brain pathways, heightening susceptibility to psychosocial stress and impairing emotional regulation and social cognition within peer settings; the result may be a premature sensitization to social threat cues that disrupts adaptive social learning, ultimately fostering maladaptive developmental pathways with consequences in long-term challenges in emotional regulation and social adaptability (34).

### 1.4 Altered timing of adrenarche and DHEA(S) levels in influencing ASD phenotypes

Despite extensive research recognizing middle childhood as a critical period for acquiring social, affective and cognitive adaptations (18, 19, 33), little focused attention has been given to how adrenarche and DHEA(S) influence these traits or how alterations during this stage may contribute to risks and expression of ASD and other manifestations of psychological maladaptation. In a longitudinal study of autistic children aged six to eleven, Waizbard-Bartov et al. (39) identified different developmental trajectories in symptom severity for ASD; children whose social-communication challenges increased tended to exhibit a simultaneous elevation in levels of anxiety, ADHD symptoms, disruptive behavior problems, and overall psychopathology, as well as reduced restricted and repetitive behavior severity related to increased anxiety. Similarly, of the two autism polygenic factors discovered by Zhang and colleagues (20), the later diagnosed autism factor had significant positive genetic correlations with internalizing disorders, trauma, and ADHD, that may be related to the expression of a late-developing, middle childhood autism subtype. Changes in DHEA(S) levels may affect its role in the amygdala’s regulation of emotional responses to external stimuli (38), heightening the fear response during social interactions and potentially reinforcing maladaptive behavior patterns during a period when enduring neural connections are being made. Overall, altered adrenarche mechanisms may thus disrupt social integration and typical cognitive development, leading to maladaptive behaviors characteristic of the autism spectrum.

## 2 Methods

### 2.1 Systematic review 1: social adaptations that arise in middle childhood and the impact of adrenarche and DHEA(S)

For each specific topic, searches for the following four systematic reviews were conducted through the online databases Web of Science, PubMed, and PsycINFO (APA PsycNET). For each review, articles were obtained based on keyword searches, the details of which are provided in **Table 1**. Abstracts were then scanned for relevance to the research question based on the inclusion and exclusion criteria for each review, as outlined below.

**Table 1:** Summary of search methods for each systematic review.

Question		Keywords	Results		Filters added	Duplicates	Didn’t meet criteria	Total articles
1(a)	Adrenarche & sociality	Adrenarch* AND social* OR “social development” OR “social cognition”	WofS: Pmed: Psinfo: Other:	59 35 26 3	43 12 1	49	570	27
1(b)	DHEA(S) & sociality	DHEA OR DHEA-S OR Dehydroepiandrosterone OR “Dehydroepiandrosterone sulfate” AND social* OR “social development” OR “social cognition”	WofS: Pmed: Psinfo: Other:	663 264 406 1	537 5 49			
2(a)	Autism & adrenarche	Autis* OR ASD AND adrenarch*	WofS: Pmed: Psinfo: Other:	7 4 5	6 1 4	10	80	14
2(b)	Autism & DHEA(S)	Autis* OR ASD AND DHEA OR DHEA-S OR Dehydroepiandrosterone OR “Dehydroepiandrosterone sulfate”	WofS: Pmed: Psinfo: Other:	79 32 77	76 2 17			
3(a)	Adrenarche & restricted interests	Adrenarch* AND intense OR focused OR special OR narrow OR circumscribed OR perseverative OR stereotyped OR interests OR preoccupations	WofS: Pmed: Psinfo: Other:	67 314 13	43 7 6	143	1866	7
3(b)	DHEA(S) & restricted interests	DHEA OR DHEA-S OR Dehydroepiandrosterone OR “Dehydroepiandrosterone sulfate” AND intense OR focused OR special OR narrow OR circumscribed OR perseverative OR stereotyped OR interests OR preoccupations	WofS: Pmed: Psinfo: Other:	1281 130 6572	921 121 21			
3(c)	Adrenarche & repetitive behaviors	Adrenarch* AND repetitive OR stereotyped OR compulsive OR habitual OR ritualistic OR behaviors OR behaviours OR movements OR gestures OR stereotypy OR perseveration OR echolalia	WofS: Pmed: Psinfo: Other:	56 38 158	44 18 5			
3(d)	DHEA(S) & repetitive behaviors	DHEA OR DHEA-S OR Dehydroepiandrosterone OR “Dehydroepiandrosterone sulfate” AND repetitive OR stereotyped OR compulsive OR habitual OR ritualistic OR behaviors OR behaviours OR movements OR gestures OR stereotypy OR perseveration OR echolalia	WofS: Pmed: Psinfo: Other:	850 3153 510	721 84 61			

Keywords were first run through each database, and the number of articles was recorded in the ‘Results’ column. Filters were then applied according to the database - in Web of Science (WoS), only journal articles and English-language publications were selected, as limited filters were available. For PsycINFO (Psinfo), the following inclusion criteria were applied: human subjects, English language, age groups (preschool [2–5 years], school age [6–12 years], adolescence [13–17 years]), and all methodologies except for qualitative studies, systematic reviews, and literature reviews. In PubMed (Pmed), the filters included: human subjects, English language, article types (adaptive clinical trial, clinical study, clinical trial, dataset, meta-analysis, randomized controlled trial), and age groups (preschool child [2–5 years], child [6–12 years], adolescent [13–18 years]). The number of articles after applying these filters were recorded under ‘Filters added.’ For each review (1a-b, 2a-b, 3a-d), results from all databases were consolidated, duplicates were removed, and abstracts were screened based on the respective inclusion and exclusion criteria.

To find all relevant literature, we performed a search for adrenarche with keywords related to social cognition, and for DHEA(S) with keywords related to social cognition and then combined the results after adding database filters (1a-b in **Table 1**). Once duplicates were removed, abstracts were screened for relevance to adrenarche or DHEA(S) in relation to social development in typical individuals. Studies were included if they provided quantitative measures of DHEA(S) relevant to their specific impacts on human social development during or around the juvenile stage (4 to 18 years of age). Bibliographies of articles fitting the search criteria were also scanned to locate additional relevant articles.

### 2.2 Systematic review 2: altered adrenarche timing and DHEA(S) levels leading to maladaptation in social cognition

Searches for adrenarche and ASD, and for DHEA(S) and ASD, were performed separately, and the results were combined after adding database filters (2a-b in **Table 1**). Once duplicates were removed, abstracts were screened for relevance to adrenarche or DHEA(S) in relation to ASD. Studies were included if they provided quantitative measures of DHEA(S) during or around the juvenile stage (4 to 18 years of age) and involved participants with an official ASD diagnosis. Bibliographies were also reviewed to locate additional relevant articles.

### 2.3 Systematic review 3: impact of DHEA(S) levels on repetitive behaviors and restricted interests

Two searches were conducted for this review: one for adrenarche and the other for DHEA(S); each was paired with a string of keywords related to repetitive behaviors. Two additional searches were performed for adrenarche and DHEA(S) with keywords related to restricted interests. The results from all four searches were combined (3a-d in **Table 1**). Once duplicates were removed, abstracts were screened for relevance to adrenarche or DHEA(S) in relation to repetitive behaviors and restricted interests. Studies were included if they provided quantitative measures of DHEA(S) in humans during or around the juvenile stage. Bibliographies were also reviewed to identify additional relevant articles.

Given the overlapping scope of the systematic reviews, some studies were included in multiple searches. For example, systematic reviews 2 and 3 overlap regarding ASD traits, leading to some articles being addressed in both reviews.

PRISMA diagrams for the reviews are provided in **Supplementary Figures 1**–**8**.

## 3 Results

### 3.1 Results of systematic review 1: social adaptations that arise in middle childhood and the impact of adrenarchal mechanisms

This section investigates how variation in DHEA and DHEAS affect emotional and social outcomes across different ages. These findings are central to the hypothesis that shifts in adrenal hormones during adrenarche may influence traits often observed in ASD, with age- and gender-specific impacts.

#### 3.1.1 Varying methods for measuring adrenarche mechanisms and psychosocial-cognitive outcomes

A summary of each study reviewed for systematic review 1 can be found in **Table 2**. Different approaches were used in measuring adrenarche mechanisms, and various psychosocial-cognitive outcomes were assessed. Twelve studies collected data on Tanner stages of pubic hair development (40–52); three studies classified children as PA using this measure, further confirmed by high DHEA(S) levels (41, 44, 49), and Shirtcliff et al. (40) examined DHEA outcomes using Tanner stage as a dependent variable. Seven studies classified PA as elevated DHEA(S) compared to age-matched peers or controlled for age when assessing the effects of DHEA(S) levels (42, 43, 47, 48, 50–52), two used specific cutoff values to split children into PA and non-PA groups (45, 53), and seven assessed DHEA as a continuous variable while controlling for age or utilizing a narrow age range (54–60). Eight studies examined the cortisol/DHEA (C/D) ratio as a marker of stress responses, given DHEA(S)’s anti-glucocorticoid properties that may counter cortisol’s neurotoxic effects, where a larger C/D ratio suggests inadequate DHEA counteraction and potential HPA axis dysregulation (46, 58, 61–66). **Table 3** provides a summary of which psychosocial-cognitive outcomes were examined and by which studies, with a summary of findings for DHEA and DHEAS.

**Table 2:** Articles selected for systematic review 1 (Q1a-b; *social adaptations that arise in middle childhood and the impact of adrenarchal mechanisms*), alongside extracted information on participant sex and age, which measurements were used to assess adrenarche and aspects of sociality, and key findings in terms of DHEA and DHEAS.

Authors DOI (Type of study)	Title	Participants	Measurements Adrenarche Sociality	
(40) Shirtcliff, E., Klimes-Dougan, B. & Marcia, S (2007). doi:10.1111/j.1469-7610.2006.01723.x (Cross-sectional)	Salivary dehydroepiandrosterone responsiveness to social challenge in adolescents with internalizing problems	213 adolescents (106 boys, 107 girls), with an average age of 13.7	Adrenal steroid hormone levels (DHEA, DHEAS, testosterone) were measured via salivary samples alongside assessments of pubertal maturation via Tanner stage criteria from parent reports.	Anxiety was reported by children via the Spence children’s anxiety scale (assesses generalized anxiety, panic, social phobia, separation anxiety, OCD, physical injury fears).
(41) Dorn et al. (2009) doi:10.1515/JPEM.2008.21.5.439 (Cross-sectional)	Differences in endocrine parameters and psychopathology in girls with premature adrenarche versus on-time adrenarche	40 girls with PA and 36 girls with on-time adrenarche, aged 6 – 8 years	Blood samples were drawn to measure cortisol, DHEAS, androstenedione, testosterone, leptin, and estradiol. Pubertal maturation was assessed via Tanner stage criteria.	Used the child behavior checklist (CBCL) to internalizing and externalizing, the teacher report form to gather teachers’ evaluations of the child’s behavior, and the diagnostic interview schedule for children to assess psychiatric disorders.
(42) Whittle et al. (2015) doi:10.1093/scan/nsv014 (Cross-sectional)	Associations between early adrenarche, affective brain function and mental health in children	40 boys and 43 girls (mean age = 9.5 yrs) who varied in adrenarchal timing and DHEA(S) levels	DHEA(S) was collected via saliva samples and physical maturation was assessed according to Tanner Stage criteria via parental report.	An emotion-viewing task was assessed in fMRI. Mental health symptoms were assessed via parent report through the child behavior checklist.
(43) Barendse et al. (2018) doi:10.1016/j.psyneuen.2018.07.020 (Cross-sectional)	Associations between adrenarcheal hormones, amygdala functional connectivity and anxiety symptoms in children	40 boys and 43 girls with a mean age of 9.5.	Measured DHEA, DHEAS, and T levels via saliva samples, assessed pubertal maturation using Tanner stages, and collected physical measures such as height and weight.	The Spence children’s anxiety scale self-report was used to measure anxiety symptoms (general anxiety, social anxiety, obsessive-compulsive, separation anxiety, panic-agoraphobia, injury anxiety). Children performed a face emotion-viewing fMRI task.
(44) Dorn et al. (1999) doi:10.1001/archpedi.153.2.137 (Cross-sectional)	Biopsychological and cognitive differences in children with premature vs. on-time adrenarche	9 children with PA (mean age 7.8 years), 20 children with on-time adrenarche (mean age 8.0 years).	Serum and saliva samples were collected to measure DHEA, DHEAS, androstenedione, estradiol, thryrotropin, and free thryoxin.	Psychological assessments were conducted using questionnaires, tests, and interviews completed by both children and parents.
(45) Ellis & Essex (2007) doi:10.1111/j.1467-8624.2007.01092.x (Longitudinal)	Family environments, adrenarche, and sexual maturation: a longitudinal test of a life history model	120 children (73 girls) along with their mothers.	Salivary concentrations of DHEA were determined, and pubertal maturation was reported by parents and children using Tanner criteria.	Parental investment and dynamics were evaluated through questionnaires, including: center for epidemiological studies-depression scale, family expressiveness questionnaire, parenting stress index.
(46) Howland et al. (2020) doi:10.1016/j.yhbeh.2020.104816 (Longitudinal)	Pubertal recalibration of cortisol-DHEA coupling in previously-institutionalized children	Children aged 7 - 15, including previously institutionalized children and non-adopted comparison children.	Salivary concentrations of DHEA and cortisol were determined, alongside clinical assessments of pubertal stage.	Children completed the modified trier social stress test for children.
(47) Mundy et al. (2015) doi:10.1016/j.jadohealth.2015.09.001 (Cross-sectional)	Adrenarche and the emotional and behavioral problems of late childhood	1,239 boys and girls aged 8 - 9	Salivary concentrations of DHEA, DHEAS and T were determined, and pubertal development was assessed using the pubertal development scale.	Emotional and behavioral problems assessed via the social difficulties questionnaire (emotional symptoms, hyperactivity/inattention, peer problems, prosociality).
(48) Murray et al. (2016) doi:10.1016/j.psyneuen.2015.11.004 (Cross-sectional)	Associations between dehydroepiandrosterone (DHEA) levels, pituitary volume, and social anxiety in children	95 girls and boys with a mean age of 9.5 years.	Serum concentrations of DHEA and DHEAS levels were determined.	Anxiety symptoms were evaluated using standardized questionnaires.
(49) Sontag-Padilla et al. (2012) doi:10.1017/S0954579411000782 (Cross-sectional)	Executive functioning, cortisol reactivity, and symptoms of psychopathology in girls with premature adrenarche	76 girls aged 6 – 8 with both early and on-time adrenarche.	Serum concentrations of DHEAS and cortisol were determined.	Executive functioning was assessed via the Wisconsin card sorting test, parents reported emotional and behavioral problems via the CBCL, and depressive and anxious symptoms were child-reported.
(50) Susman et al. (1985) doi:10.1007/BF02090322 (Cross-sectional)	The relation of relative hormonal levels and physical development and social-emotional behavior in young adolescents	56 boys and 52 girls aged 9 - 14.	Serum concentrations of luteinizing hormone, follicle stimulating hormone (FSH), testosterone (T), estradiol, DHEA, DHEAS, and androstenedione were determined. Physical maturation was assessed via Tanner criteria.	Family and social relationships were assessed via interviews and the offer self-image questionnaire for adolescents assessed adjustment in family and peer relationships.
(51) Barendse et al. (2022) doi:10.1037/abn0000721 (Longitudinal)	Multimethod assessment of pubertal timing and associations with internalizing psychopathology in early adolescent girls	174 female adolescents aged 10 – 13 years.	Pubertal timing was measured by participants via the pubertal development scale, and DHEA was assessed through saliva samples.	Mental health outcomes were evaluated using standardized depression and anxiety symptom scales at two time points over 18 months.
(52) Barendse et al. (2020) doi:10.1016/j.jaac.2019.04.018 (Longitudinal)	Adrenarcheal timing longitudinally predicts anxiety symptoms via amygdala connectivity during emotion processing	34 girls and 30 boys with a mean age of 9.5 at time 1 (T1) and a mean age of 12.2 at time 2 (T2).	Salivary concentrations of T, DHEA, and DHEAS were determined.	Obtained fMRI scans to assess amygdala connectivity during fearful emotional processing. To assess anxiety symptoms, participants completed the Spence children’s anxiety scale.
(53) Mäntyselkä et al. (2019) doi:10.1159/000501719 (Cross-sectional)	Associations of IGF - 1 and adrenal androgens with cognition in childhood	183 girls and 204 boys aged 6 – 8.	Serum concentrations of DHEAS, androstenedione, testosterone, and IGF - 1 were measured.	Raven’s coloured progressive matrices assessed nonverbal reasoning (tests executive functioning: inhibition, working memory, cognitive flexibility) was used.
(54) Mulligan et al. (2020) doi:10.1016/j.psyneuen.2020.104751 (Cross-sectional)	Increased dehydroepiandrosterone (DHEA) is associated with anxiety in adolescent girls	286 adolescent girls aged 8 – 14 with a mean age of 12.6 years.	Salivary concentrations of DHEA, T, estradiol, and progesterone were obtained, and the pubertal development scale measured pubertal maturation via self-report.	The screen for child anxiety related disorders was used to determine measures of self-reported anxiety, and the kiddie schedule for affective disorders and schizophrenia assessed for psychopathology.
(55) Pajer et al. (2006) doi:10.1016/j.psyneuen.2006.09.005 (Cross-sectional)	Adrenal androgen and gonadal hormone levels in adolescent girls with conduct disorder	87 girls aged 15 - 17, including 47 with conduct disorder and 36 controls.	Hormones including T, estradiol, DHEA, DHEAS, cortisol, and SHBG were measured from blood samples.	Used a structured psychiatric interview to categorize girls into conduct disorder and non-conduct disorder groups.
(56) Azurmendi et al. (2006) doi:10.1016/j.yhbeh.2006.02.004 (Longitudinal)	Aggression, dominance, and affiliation:: Their relationships with androgen levels and intelligence in 5-year-old children	129 preschool children (60 boys, 69 girls), with a mean age of 5 years old.	Salivary DHEA, T, and androstenedione levels were determined.	Social interactions were observed during free play sessions, focusing on behaviors related to aggression, affiliation, and dominance, analyzed via video recordings.
(57) Cardoos et al. (2017) doi: (Cross-sectional)	Social status strategy in early adolescent girls: Testosterone and value-based decision making	63 girls aged 10 - 14.	Salivary concentrations of T, DHEA, and estradiol were determined.	Evaluated social motivation and risk-taking behavior via an auction task (assesses willingness to take financial risks for social status).
(58) Marceau et al. (2014) doi:10.1016/j.psyneuen.2013.12.002 (Cross-sectional)	Within-adolescent coupled changes in cortisol with DHEA and testosterone in response to three stressors during adolescence	213 boys aged 11 – 16 with a mean age of 13.7, and 108 girls aged 9 – 14 with a mean age of 12.0.	Hormone levels of DHEA, cortisol and testosterone were assayed from saliva and serum samples.	3 laboratory-based stressors: parent-adolescent conflict discussion paradigm, social performance paradigm, venipuncture paradigm. Participants completed a conflict discussion paradigm and a social performance paradigm.

Studies incorporating neuroimaging alongside other social behavior measures are listed separately at the bottom of the table.

**Table 3A:** Summary of findings for internalizing and externalizing, organized in order of age range for each trait.

Trait	Study	Age range (mean)	Sex	Internalizing/externalizing outcome	DHEA levels	DHEAS levels
Internalizing (e.g., anxiety, depression, somatic complaints)	(49) (41) (44) (63) (40) (61)	6-8 (7.65) 6-8 (7.7) 6-9 (7.9) 8-12 (10.4) 11-13 (-) 14-16 (-) 13-16 (14.9)	F F M/F M/F M/F M/F M/F	I I I I ns I (F) ns	- - I (PA) I (C/D) ns D -	I (PA) I (PA) I (PA) - - - ns
Anxiety	(41) (44) (48) (43) (52) (51) (54) (66)	6-8 (7.7) 6-9 (7.9) 9 (9.5) 9 (9.53) 9-12 (10.9) 10-13 (12.4) 8-14 (12.6) 12-16 (14.4)	F M/F M/F M/F M/F F F F	I ns I (indirectly) D (F)/I (M) (indirectly) D (F)/I (M) (indirectly) ns I ns	- ns I I I ns I ns	I (PA) ns I I ns - - -
Depression	(49) (44) (51) (62) (64)	6-8 (7.65) 6-8 (7.9) 10-13 (12.4) 11-13 (13.2) 12-16 (13.9)	F F F F M/F	ns I ns I I (depression risk)	- I (PA) ns I (C/D) I (C/D)	ns I (PA) - - -
Externalizing (e.g. risk-taking, aggression, delinquent behavior)	(41) (49) (44) (42) (63) (40) (61)	6-8 (7.7) 6-8 (7.65) 6-9 (7.9) 9 (9.53) 8-12 (10.4) 11-16 (13.7) 13-16 (14.9)	F F M/F M/F M/F M/F M/F	I I I I (F) ns ns I	- - I (PA) I ns ns -	I (PA) I (PA) I (PA) - - - D (C/D)
Aggression	(56) (47) (63) (50) (55)	5 (5.5) 8-9 (9.0) 8-12 (10.4) 9-14 (12.4) 15-17 (16.5)	M/F M/F M/F M/F F	ns I (M) ns ns ns	ns ns ns ns ns	- I - ns ns
Hyperactivity/inattention	(41) (44) (47)	6-8 (7.7) 6-9 (7.9) 8-9 (9.0)	F M/F M/F	I ns (attention) I (M)	- ns ns	I (PA) ns I

(C/D) next to results indicates that a significant relationship was found only for cortisol/DHEA(S) ratios. Whether the study focused on boys or on both boys and girls is specified in the ‘Sex’ column. (M/F) indicates that sex-differences were found for DHEA(S) levels in relation to psycho-social cognitive outcomes. I, increased; D, decreased; ns, no relationship.

**Table 3B:** Summary of findings for social outcomes.

Trait	Study	Age range (mean)	Sex	Social outcome	DHEA levels	DHEAS levels
Prosociality & social motivation	(56) (44) (47) (50) (57)	5 (5.5) 6-9 (7.9) 8-9 (9.0) 9-14 (12.4) 10-14 (12.7)	M/F M/F M/F M/F F	ns ns ns I (M; interest in dating only) ns	ns ns ns I ns	- ns ns - -
Social adversity (family/peer problems, trauma)	(59) (60) (45) (41) (47) (62) (66) (61)	0-5 (1.25) 1-11 (6.3) 6-7 (7.25) 6-8 (7.7) 8-9 (9.0) 11-13 (13.2) 12-16 (14.4) 13-16 (14.9)	M/F M/F M/F F M/F F F M/F	ns (family dysfunction) ns (cumulative social adversity) I (family conflict)/D (support) I (social problems) I (peer problems) I (disappointing life events) D (maternal confiding, support) ns (deprivation)	I ns I (M) - I (M) I (C/D) I -	- - I I (PA) I - - ns
Social stress response	(46) (58) (40) (64) (65) (61)	7-15 (11.4) 9-16 (13.7) 11-16 (13.7) 12-16 (13.9) 12-16 (14.0) 13-16 (14.9)	M/F M/F M/F M/F M/F M/F	I (post-stress C/D coupling) I I (response to stress tasks) I I (response to stress tasks) I (interpersonal violence)	I (C/D) I I I (C/D) I -	- - - - - D (C/D)

**Table 3C:** Summary of findings for cognitive outcomes.

Trait	Study	Age range (mean)	Sex	Cognitive outcome	DHEA levels	DHEAS levels
Executive functioning	(53) (49) (44) (62)	6-8 (7.6) 6-8 (7.65) 6-8 (7.9) 11-13 (13.2)	M/F F M/F F	ns D D D (memory function)	- - I (PA) I (C/D)	ns I (PA) I (PA) -

#### 3.1.2 The impact of DHEA, DHEAS and premature adrenarche on internalizing and externalizing traits

Elevated DHEA levels were generally associated with increased internalizing and externalizing symptoms compared to TD peers, though effects varied by age and gender. Barendse et al. (51) found no direct association between levels of DHEA and internalizing symptoms in girls aged 10-13; however, internalizing was associated with earlier pubertal maturation. Mulligan et al. (54) observed that heightened DHEA levels in girls aged 8–14 were associated with higher levels of anxiety, particularly increased panic/agoraphobia, generalized anxiety, and social anxiety. In girls and boys aged 8-12, average cortisol and high DHEA levels were related to increased internalizing (63). DHEA was not related to internalizing or externalizing in girls or boys aged 11-13, however, low DHEA levels were linked to increased internalizing symptoms in girls aged 14-16 (40). Goodyer et al. (62) found that elevated evening cortisol and decreased morning DHEA levels and high cortisol/DHEA (C/D) ratios predicted the persistence of major depression in adolescents. Additionally, in boys aged 9-14, higher DHEA levels were linked to increased sad affect and emotional difficulties (50). Pajer et al. (55) found no differences in DHEA in girls aged 15–17 with conduct disorder compared to controls, though they had lower C/D ratios, and girls with aggressive conduct disorder exhibited even lower ratios compared to those with non-aggressive conduct disorder.

Levels of DHEAS showed variable associations with internalizing and externalizing traits, as well as with cognitive outcomes. In comparison to girls with on-time adrenarche, girls aged 6–8 with premature adrenarche (as inferred by high age-specific DHEAS levels) displayed increased internalizing and externalizing, with higher symptom scores for separation anxiety, specific phobia, panic/agoraphobia, depression, attention-deficit hyperactivity disorder (ADHD), aggression, oppositional defiant disorder (ODD), obsessive-compulsive disorder (OCD) and social problems (41). Sontag-Padilla et al. (49) found that girls with PA aged 6–8 with low executive functioning exhibited higher internalizing and externalizing; however, these traits were not significant in girls with high executive functioning. Dorn et al. (44) also found increased internalizing and externalizing in girls and boys aged 6–9 with PA (confirmed by increased DHEA and DHEAS), with higher scores on withdrawal, somatic complaints, aggression, and social problems; they further reported lower scores for cognitive skills, encompassing verbal comprehension, perceptual organization, freedom from distractibility, and processing speed. In contrast, Mäntyselkä et al. (53) found no relationship between DHEAS and cognitive measures (abstract reasoning, pattern recognition, problem-solving skills) in boys and girls aged 6-8.

Neuroimaging studies have also reported evidence salient to relationships of DHEA and DHEAS with internalizing and externalizing traits. Thus, in nine-year-old girls and boys, high DHEA levels were indirectly related to increased social anxiety and obsessive-compulsive symptoms through increased pituitary gland volume (48). Barendse et al. (43) reported indirect, gender-specific effects of elevated DHEA and DHEAS in nine-year-old children, with both boys and girls exhibiting reduced connectivity between the right amygdala and bilateral cerebellum; boys showed heightened social anxiety and obsessive-compulsive symptoms associated with increased left amygdala connectivity to the visual cortex, while girls with elevated DHEAS displayed reduced connectivity between the right amygdala and the right fusiform gyrus (FFA) and left insula, which was linked to decreases in social anxiety, specific phobia, and generalized anxiety. Whittle et al. (42) observed reduced activation in the mid-cingulate cortex in response to emotional faces in nine-year-old girls and boys; however, externalizing symptoms were found only in girls (internalizing was not assessed) and were linked to decreased posterior insula but increased ventromedial prefrontal cortex (vmPFC) activation when viewing happy faces; girls also exhibited reduced activation in the subgenual cingulate cortex to happy faces and reduced activation in the dorsolateral prefrontal cortex (dlPFC) and striatum in response to negative emotional faces. In boys, elevated DHEA levels at age nine were associated with increased amygdala connectivity with lateral prefrontal areas and the anterior cingulate cortex (ACC) by age twelve, while elevated DHEAS levels were linked to increased amygdala-inferior frontal gyrus (IFG) connectivity over time, both of which were associated with increased anxiety symptoms; in contrast, girls with elevated DHEAS levels exhibited decreased amygdala-IFG connectivity over time, which was associated with reduced anxiety symptoms, and no significant differences in amygdala connectivity were observed in relation to changes in hormone levels (adrenarchal tempo) (52).

In summary, of twelve studies that examined internalizing, seven found associations between elevated DHEA or DHEAS levels and increased internalizing symptoms (41, 43, 44, 49, 52, 54, 63), Barendse et al. (51) reported no association, Shirtcliff et al. (40) found an inverse relationship in older adolescents, and three studies found increased C/D ratios (62–64). Out of nine studies that examined externalizing, six reported increased externalizing traits associated with elevated DHEA or DHEAS (41, 42, 44, 47–49), Shirtcliff et al. (40) found no association, and Pajer et al. (55) noted no differences in DHEAS levels between conduct disorder cases and controls but an increased C/D ratio.

#### 3.1.3 The impact of DHEA, DHEAS and premature adrenarche on social outcomes

Studies that assessed social outcomes in relation to DHEA reported varied outcomes. Girls exhibited higher DHEA levels and engaged more frequently in prosocial behaviors in comparison to boys; however, no significant relationship was found between DHEA and prosocial behavior in five-year old girls or boys (56), or for girls or boys aged 8-9 (47). Similarly, assessments of social dominance behaviors showed no associations with DHEA levels in five-year-old girls or boys (56) or in girls aged 10-14 (57). Girls aged 11–13 with high C/D ratios reported more negative life events (62), and Shirtcliff et al. (40) found that 11-16-year-old girls and boys, particularly girls with internalizing problems, exhibited a heightened DHEA response to a peer evaluation task, both in a laboratory and naturalistic setting (diurnal rhythm), with internalizing associated with more negative life events, rumination, and emotion-focused coping. Howland et al. (46) did not find any differences in DHEA levels in previously institutionalized girls and boys aged 7–15 compared to controls, though they found positive C/D coupling, where high cortisol levels coincide with high DHEA levels, in both groups. However, in contrast to non-adopted children, there was no C/D coupling in early adrenarchal stages, but cortisol and DHEA became coupled in later pubertal stages. In girls and boys aged 9-16, a positive C/D coupling was observed in response to a social performance task, with older adolescents exhibiting stronger coupling (58). While elevated DHEA levels alone were linked to a more typical cortisol response to trauma-induced stress, higher DHEA/cortisol ratios were associated with a stronger link between childhood maltreatment and reduced cortisol responses, suggesting an adrenal-specific adaptation (65). Lastly, Pantell et al. (60) found no relationship between DHEA and cumulative social adversity in boys aged 0-17.

Social-emotional challenges related to DHEA may also extend to early adversity. High DHEA levels were associated with socioeconomic disadvantage, indicating early adversity; however, no relationship was found with family dysfunction, in girls and boys aged 0-5 (59). Lower levels of parental supportiveness and higher levels of father-reported marital conflict and depression during the preschool years predicted PA in both boys and girls aged 6-7, and mother’s age at menarche and socioeconomic status (SES) predicted the timing of sexual development in daughters at age eleven (45). High cortisol and low DHEA levels were linked to increased emotional regulation and likability among peers and adults in both sexes aged 8-12, despite higher maltreatment in girls, with girls having higher DHEA levels compared to boys (63). In addition, blunted (decreased) HPA-axis reactivity, reflected in lower cortisol/DHEAS ratios, was observed in girls and boys aged 13–16 exposed to greater interpersonal violence, and was found to mediate the positive association between such exposure and increased externalizing psychopathology (61). Cardoos et al. (57) found no relationship between DHEA and SES in girls aged 10-14; however, SES positively predicted risk-taking behavior. In girls and boys aged 9-16, Marceau et al. (58) observed increased C/D coupling in response to a parent-child conflict task, however Shirtcliff et al. (40) reported reduced DHEA levels in girls and boys aged 11-16. High DHEA levels were associated with less confiding in one’s mother and less maternal support in girls aged 12-16, and lower DHEA levels were initially linked to higher attachment anxiety, though this relationship lost significance after controlling for cortisol (66).

Of thirteen studies that examined social outcomes in relation to DHEA, DHEAS, or C/D ratios, five found elevated levels in relation to negative social outcomes, including negative life events, maltreatment, or family conflict (40, 45, 58, 62, 65). Five studies found no association between DHEA levels and prosocial or dominance behaviors (46, 47, 56, 57) or with cumulative social adversity (60). Two studies linked high DHEA levels to socioeconomic disadvantage (45, 59), whereas Cardoos et al. (57) observed no relationship between DHEA and SES.

Overall, elevated DHEA and DHEAS levels were associated with increased internalizing and externalizing symptoms, although these effects varied by age, gender, and context. Neuroimaging studies also showed that heightened DHEA and DHEAS levels were related to changes in brain connectivity, influencing emotional and behavioral traits like anxiety and externalizing behaviors. Studies on DHEA and DHEAS and social outcomes showed mixed results, with no relationship found between DHEA or DHEAS and prosociality or social competence; however, increased responsivity was found in response to social stress tasks.

### 3.2 Results for systematic review 2: altered adrenarche timing and DHEA(S) levels in relation to social cognition

This section investigates how alterations in adrenarche timing and DHEA(S) levels may contribute to the manifestation of ASD by examining whether DHEA(S) levels differ in ASD children compared to neurotypical children, as well as the relationships with DHEA(S) and social impairments in ASD, specifically focusing on androgenic enzyme activity and sex-based differences.

#### 3.2.1 Different sampling methods

A summary of each study reviewed for systematic review 2 can be found in **Table 4**. Different methods were utilized between studies in measuring DHEA(S) and in assessing ASD. Six studies measured only DHEA or DHEAS (67–73) and five measured both (74–78). In eight studies, children were diagnosed or had a previous diagnosis based on DSM-IV criteria (67–71, 76, 77, 79), based on DSM-5 criteria in three studies (72, 74, 78), based on ICD-10 or DSM-III-R criteria by Lakatošová et al. (75), and Tordjman et al. (73),, respectively, and Geier & Geier (80) did not specify. Nine studies evaluated only boys with ASD (67–69, 71, 72, 74–76, 78), with five evaluating both girls and boys (70, 73, 77, 79, 80). Six studies provided measures of ASD social impairments (69, 71–75), and three examined the association between DHEA(S) levels and autism severity as measured by the Childhood Autism Rating Scale (CARS) (69, 77, 78).

**Table 4:** Articles selected for review for systematic review 2 (Q2a-b; *altered adrenarchal timing and DHEA(S) levels leading to maladaptation in social cognition*), alongside extracted information on participant sex and age, which measurements were used to assess adrenarche and diagnose ASD and/or related traits, and key findings in terms of DHEA and DHEAS.

Authors DOI (Type of study)	Title	Participants	Measurements Adrenarche ASD traits		Key findings
(67) Al-Zaid et al. (2021) doi:10.1038/s41598-021-97266-8 (Cross-sectional)	A potential role for the adrenal gland in autism	31 ASD boys and 28 healthy, age-matched controls.	Obtained blood samples to measure estradiol, DHEA, FSH, total testosterone (TT), FT and luteinizing hormone (LH).	Children met the DSM-IV criteria for autism diagnosis previously determined using the autism diagnostic observation schedule and CARS.	Significantly elevated levels of DHEA and FSH in boys with ASD. A strong positive correlation was found between the elevated levels of TT and FT with DHEA levels. Lower 2^nd^-to-4^th^ digit ratio has been reported in the same ASD group.
(68) Croonenberghs et al. (2008) doi: (Cross-sectional)	Faulty serotonin - DHEA interactions in autism: results of the 5-hydroxytryptophan challenge test	18 ASD boys matched with 22 healthy controls, aged 13 – 19 years old.	Plasma DHEAS and cortisol levels were measured before and after administration of 5-HTP or placebo.	Diagnosis was confirmed via DSM-IV criteria through the autism diagnostic observation schedule. Parents completed the child behavior checklist and aberrant behavior checklist.	ASD children had a higher baseline cortisol/DHEAS ratio and a higher 5-HTP-induced DHEAS response. Thought problems, social problems, withdrawal, somatic complaints, anxiety, depression, attention problems were significantly greater in ASD group. An additional measure also supported increased internalizing (withdrawal, attention problems).
(69) El-Baz et al. (2014) doi:10.1515/ijamh-2012-0116 (Cross-sectional)	Hyperandrogenemia in male autistic children and adolescents: Relation to disease severity	30 ASD male children and adolescents, with a mean age of 9.13 years, and 20 healthy age- and sex-matched controls.	Serum samples were obtained to collect measures of FT, DHEA, and Δ4-A, and pubertal maturation was assessed according to Tanner criteria.	Diagnoses were confirmed using DSM-IV criteria. ASD severity was assessed via the childhood autism rating scale (CARS).	Androgen levels were higher in autistic patients than in controls and increased with disease severity. FT and Δ4-A had a significant risk for association with autism; ASD risk was non-significant for DHEA. Hyperandrogenemia is prevalent in ASD boys; autism severity, as well as levels of FT and DHEA increased with increasing Tanner pubertal stages. DHEA and FT positively correlated with CARS scores.
(70) Gasser et al. (2022) doi:10.3390/life12060867 (Cross-sectional)	How is CYP17A1 activity altered in autism? A pilot study to identify potential pharmacological targets	48 ASD boys (mean age 14.2 years), 48 healthy age-matched boys, 16 ASD girls (mean age 13.8 years),16 healthy age-matched girls.	Urine samples were collected to obtain measures for steroid hormone metabolites, including those for DHEA, cortisol, androstendione, and T.	Children had previous ASD diagnoses given in the first years of life according to the diagnostic criteria of the DSM-IV and confirmed by a clinician.	Increased activity of 17-alpha Hydroxylase, 17/20 Lyase in ASD children. Altered steroid hormone metabolites with increased levels of androgens and glucocorticoids. The ratios cortisol/DHEA, cortisol/androstenedione, and cortisol/testosterone were also altered. DHEA and T was higher in girls, and androstendione was higher in boys. Average metabolite levels higher in ASD.
(71) Hassan et al. (2019) doi:10.1007/s12031-018-1225-9 (Cross-sectional)	Possible metabolic alterations among autistic male children: Clinical and biochemical approaches	146 boys aged 4 - 11, divided into 73 males with autism and 73 healthy age- and sex-matched children.	Serum concentrations of markers of mitochondrial dysfunction, oxidative stress, and heavy metals, including DHEA, cortisol, and free T, were determined.	Neuropsychological assessments, including CARS, short sensory profile, and intelligent quotients, were conducted.	Evidence of mitochondrial dysfunction, low cholesterol levels in ASD boys. Significantly lower total cholesterol, cortisol, and estradiol levels, as well as higher DHEA and free testosterone levels.
(72) He et al. (2023) doi:10.1186/s12888-023-04586-2 (Cross-sectional)	Analysis of salivary steroid hormones in boys with autism spectrum disorder	55 boys with ASD and 24 neurotypical controls.	Saliva samples were collected to obtain measures of cortisol, DHEA, and pregnenolone.	Utilized the child behavior checklist, autism behavior checklist, social responsiveness scale, and the repetitive behavior scale to assess ASD symptom severity.	Pregnenolone but not DHEA was associated with the RBS score, and boys with higher pregnenolone had a higher risk of ASD. Only pregnenolone was linked to autism behavior checklist scores.
(73) Tordjman et al. (1995) doi:10.1007/BF02179290 (Cross-sectional)	Plasma androgens in autism	39 autistic subjects (31 prepubertal, 8 postpubertal), 12 cognitively impaired subjects (all prepubertal), and 21 normal control subjects (10 prepubertal, 11 postpubertal).	Serum concentrations of DHEAS, testosterone (T), and whole blood serotonin (5-HT) were determined, and pubertal maturation was assessed via Tanner Stage criteria.	Behavioral assessments via the autism behavior checklist, autism diagnostic observation schedule, Vineland adaptive behavior scales, and a scale for lifetime aggression, which test for autism severity, self-injurious behaviour, and stereotypies.	No correlations with DHEAS and autism severity, self-injurious behavior, aggression, or stereotypies.
(74) Jansáková et al. (2020) doi:10.1038/s41398-020-01017-8 (Cross-sectional)	Alteration of the steroidogenesis in boys with autism spectrum disorders	62 prepubertal boys with ASD and 24 age- and sex-matched neurotypical controls.	Plasma samples were collected to obtain 82 biomarkers of steroidogenesis, including DHEA, DHEAS, progesterone.	Children were diagnosed using the DSM-IV criteria through the Autism Diagnostic Observation Schedule, 2nd revision (ADOS - 2), and the Autism Diagnostic Interview-Revised (ADI-R).	Correlations were found between behavioral indices and circulating steroids, with the best correlation found for social interaction. DHEA and DHEAS were both found to be strong predictors of ASD subdomains: social interaction, communication and language, and repetitive, restricted and stereotyped interests.
(75) Lakatosová et al. (2022) doi:10.30773/pi.2021.0094 (Cross-sectional)	The relationship of steroid hormones, genes related to testosterone metabolism and behavior in boys with autism in Slovakia	172 boys with a mean age of 5.2 years of age, and 135 neurotypical boys with a mean age of 11.	Plasma samples were obtained to determine the concentration of testosterone, estradiol, DHEA, DHEAS, and sex hormone binding globulin (SHBG).	Boys underwent behavioral testing via ADI-R and ADOS - 2 to evaluate the core symptom severities of ASD, the behavior problem inventory questionnaire assessed stereotyped behavior, aggression, self-injurious behaviors.	SHBG, DHEA, and DHEAS positively correlated with age. Negative relationship between SHBG levels and restricted, repetitive behaviors, and a positive relationship between testosterone levels and frequency of stereotyped behavior. SHBG levels and testosterone are related to the severities of restricted and repetitive behaviors in boys.
(76) Mills et al. (2007) doi:10.1111/j.1365-2265.2007.02868.x (Cross-sectional)	Elevated levels of growth‐related hormones in autism and autism spectrum disorder	71 boys aged 4 – 8 with ASD and 59 age-matched controls.	Levels of IGF - 1, IGF - 2, IGFBP - 3, GHBP, DHEA, and DHEAS were acquired from blood samples.	Children with a previous ASD diagnosis according to DSM-IV criteria were recruited and confirmed through the autism diagnosis observation schedule.	ASD children had significantly greater head circumferences, weights, BMIs, and higher levels of IGF - 1, IGF - 2, IGFBP - 3, and GHBP. Mean levels of DHEA or DHEAS were not different between ASD and controls. ASD boys are more likely to have detectable DHEAS levels; higher levels were found but were not significant. DHEA and DHEAS positively correlated with weight, height, and head circumference; only DHEA and weight correlated in controls. Those with measurable DHEAS had higher IGF - 1 levels.
(77) Majewska et al. (2014) doi:10.1007/s00787-013-0472-0 (Cross-sectional)	Marked elevation of adrenal steroids, especially androgens, in saliva of prepubertal autistic children	78 prepubertal autistic male and female children from two age groups (3 – 4 and 7 – 9 years old) along with 70 neurotypical controls.	Salivary concentrations of 22 steroids, including androstenediol, DHEA, DHEAS, DHEA - 7o, pregnenolone, and androsterone, were determined.	Each child was rediagnosed by clinicians according to the DSM–IV criteria for ASD, and severity of traits was assessed according to the childhood autism rating scale (CARS).	More abnormal development (e.g. fine motor skills, speech delay, hypotonia, poor sleep) and hyperactivity was found in ASD children. In older ASD girls, CARS scores correlated positively with multiple steroids, including DHEA, DHEAS, DHEA - 7o, androstenediol, but older boys exhibited weak negative correlations with DHEA and DHEAS only. In younger ASD girls, CARS scores negatively correlated with androsterone, DHEA - 7o, and DHEA.
(78) Sriram et al. (2022) doi:10.1007/s12098-022-04080-9 (Cross-sectional)	Evaluation of hyperandrogenism in children with autism spectrum disorder	32 children with ASD (mean age: 8.5 years) and 23 healthy controls of similar age, sex, and Tanner stage.	Plasma samples were obtained for measures of T, DHEAS, and androstenedione, and pubertal maturation was assessed via Tanner criteria.	Children were previously diagnosed with ASD according to DSM - 5 criteria, and underwent an ASD severity assessment using the CARS.	There was no significant difference in androgen levels in the two groups. T levels positively correlated with frequency of stereotyped behavior. Among children with ASD, elevated testosterone, DHEAS, and androstenedione levels were seen in 12.3%, 6.2%, and 9%, respectively. No significant correlation between ASD severity and levels of T, DHEAS, and androstenedione.
(79) Geier & Geier (2007) doi: (Cross-sectional)	A prospective assessment of androgen levels in patients with autistic spectrum disorders: biochemical underpinnings and suggested therapies	70 ASD male and female children aged 7 - 15.	Serum concentrations of T, free testosterone (FT), DHEA, androstendione, and follicle-stimulating hormone (FSH) were determined.	Each child had been previously diagnosed with ASD according to DSM-IV criteria.	Increased serum T, serum FT, % FT, DHEA, and androstenedione in ASD children. No sex differences for DHEA or androstenedione, but females had higher levels of T. 81% of patients had at least one androgen marker greater than the upper limit for their age- and sex-specific reference range. Significantly increased androgen metabolites in patients with ASD, and significantly reduced FSH levels.
(80) Geier & Geier (2006) doi:10.1159/000094467 (Cross-sectional)	A clinical and laboratory evaluation of methionine cycle-transsulfuration and androgen pathway markers in children with autistic disorders	16 ASD pre-pubertal girls and boys aged 4 - 8.	Clinical exams evaluated hyperandrogenicity (early growth spurt, increased body/facial hair, aggression, early secondary sexual changes). Serum concentrations of T, DHEA, FSH and metabolites were determined.	Each child had been previously diagnosed with ASD according to DSM-IV criteria.	Increased levels of DHEA and total T, and decreased levels of FSH, reduced glutathione, cysteine, methionine, cystathionine, and homocysteine in ASD children. 94% had one or more hyperandrogenicity symptoms (aggression, increased body/facial hair, early growth spurt, early secondary sexual changes).

#### 3.2.2 Altered DHEA and DHEAS levels in children with ASD

Findings of DHEA levels in children with ASD varied by sex and age; whether DHEA and DHEAS levels in autistic boys and girls are elevated compared to controls or reference ranges is summarized in **Table 5**. Among studies examining only boys with ASD, four reported elevated DHEA levels in comparison to neurotypical controls in five-year-olds (67, 75), in boys aged 4-5 (72) and in boys aged 3-10 (71). In contrast, three found no differences in DHEA levels in ASD boys aged 3-5 (74) or in boys aged 4–8 (76). El-Baz et al. (69) found increased hyperandrogenemia, defined as androgen levels (including DHEA) exceeding two standard deviations above age- and pubertal stage-matched reference ranges, and more advanced Tanner stages in ASD boys aged 6-15; however, DHEA was not significantly associated with presence *vs* absence of ASD. Of studies that examined both boys and girls, three found elevated DHEA in girls and boys aged 4-8 (80), 7-15 (79), and 13-14 (70). Geier & Geier (2006) also found increased hyperandrogenicity in ASD girls and boys, with 94% exhibiting one or more signs. Majewska et al. (77) found that DHEA levels increased significantly in ASD between the ages 3–4 and 7–9 in girls and boys, whereas levels decreased in neurotypical controls.

**Table 5:** Summary of DHEA(S) findings in ASD children from each study, organized in order of age range.

Study	Age range (mean)	Sex	DHEA levels	DHEAS levels
(74)	3-5 (4.4)	M	ns	D
(72)	4-5 (5.17)	M	I	–
(75)	5 (5.19)	M	ns	ns
(73)	3-8 (5.55)	M	–	ns
(67)	5 (5.60)	M	I	-
(80)	4-8 (5.9)	M/F	I	–
(76)	4-8 (6.6)	M	ns	ns
(71)	3-10 (7.13)	M	I	–
(77)	7-9 (8.2)	M/F	I	I (M)
(78)	8-9 (8.5)	M	–	ns
(69)	6-15 (9.45)	M	ns	-
(79)	7-15 (10.8)	M/F	I	–
(70)	13-14 (14.2)	M/F	I	-
(68)	13-19 (-)	M	–	I

Whether the study focused on boys or on both boys and girls is specified in the ‘Sex’ column. I, increased; D, decreased; ns, no relationship.

Findings of DHEAS levels in children with ASD also show notable variation among studies. Of studies that only examined boys, five found no differences in DHEAS levels in 5-year-old boys with ASD (75) or in ASD boys aged 3-5 (74), 3-8 (73), or 8–9 (78); Mills et al. (76) found no difference in mean DHEAS levels in ASD boys aged 4–8 compared to controls, however ASD boys were more likely to have detectable levels of DHEAS, suggesting potential earlier adrenal activation. Croonenberghs et al. (68) reported higher 5-HTP-induced DHEAS levels and greater CD ratios in ASD boys aged 13-19. Lastly, for studies that assessed both girls and boys, Majewska et al. (77) found that in comparison to 3-4-year-old girls and boys with ASD, boys aged 7-9 (but not girls) exhibited increased DHEAS compared to neurotypical controls, finding that DHEAS levels increased between the two age groups in both ASD and neurotypical girls and boys. Geier & Geier (79) found lower DHEAS levels in ASD boys aged 7–15 compared to laboratory age- and sex-specific reference ranges.

Some studies yielded findings suggesting alterations in androgen-related pathways involving DHEA(S) in boys with ASD. In the ‘frontdoor’ androgen pathway, pregnenolone is first converted to 17-OH pregnenolone by CYP17A1, and then to DHEA by the same enzyme through its 17/20 lyase activity; Jansáková et al. (74) found increased DHEA/pregnenolone and DHEAS/pregnenolone-sulfate ratios in autistic boys aged 4–6 compared to neurotypical controls, suggesting heightened 17,20-lyase activity. In addition, they found increased SULT2A1 activity, which sulfates DHEA to DHEAS, which can then be converted back to DHEA; increased activity could contribute to a higher availability of DHEA as a substrate for CYP17A1. While Gasser et al. (70) found higher 17,20-lyase activity of CYP17A1 based on metabolite ratios, around 150% higher in boys with ASD, as well as a higher DC ratio, suggesting a trend towards increased DHEA production over cortisol. Geier & Geier (80) found that DHEA levels were almost 200% higher in ASD girls and boys compared to laboratory-specific age-matched reference ranges, and found reduced levels of glutathione, a peptide that hinders the conversion of DHEA to DHEAS, potentially leading to the production of less DHEAS in comparison with DHEA. This inference is supported by their later findings of lower DHEAS in boys and girls aged 7-15 (79). Four out of seven studies assessing DHEAS found no differences in levels compared to controls, and of these studies, six examined only boys.

In sum, results indicate a pattern of elevated DHEA levels in ASD compared to neurotypical controls, with seven out of eleven studies finding statistically significant increases, and eight studies assessing DHEA examined only boys. DHEAS levels are consistently not significant, with four out of seven studies that assessed DHEAS finding no differences compared to neurotypical controls, and six studies assessing DHEAS examined only boys.

#### 3.2.3 The impact of DHEA and DHEAS on ASD social interaction differences and trait severity

Most studies found no relationship between DHEA(S), social interaction differences, and ASD trait severity (see **Table 6** for a summary). Janšáková et al. (74) found a significant positive relationship of DHEA and DHEAS with reciprocal social interaction (social affect) and communication (core domains assessed via the Autism Observation Schedule) in ASD boys aged 4-6. Three studies found no relationship between DHEA and social communication differences in five-year-old boys (75), in boys aged 4-5 (72), or in boys aged 6-15 (69). Additionally, neither Hassan et al. (71) or Tordjman et al. (73) found significant associations between DHEA or DHEAS, respectively, and social communication impairments in boys aged 3-8. Of studies that assessed ASD severity using the CARS, El-Baz et al. (69) found a positive correlation with DHEA in ASD boys aged 7-16, and Sririam et al. (78) found no relationship with DHEAS in boys aged 8-9. Girls and boys aged 7–9 with ASD exhibited relationships with both DHEA and DHEAS; significant moderate positive correlations were found for girls, whereas non-significant weak negative correlations were found for boys (77).

**Table 6:** Summary of DHEA(S) impacts on ASD traits from each study, organized in order of age range.

Study	Age range (mean)	Sex	Social interaction differences	ASD trait severity	RRBs	DHEA levels	DHEAS levels
(74)	3-5 (4.4)	M	I	-	I	I	I
(72)	4-5 (5.17)	M	ns	–	ns	ns	–
(75)	5 (5.19)	M	ns	-	ns	ns	ns
(73)	3-8 (5.55)	M	ns	–	ns	–	ns
(71)	3-10 (7.13)	M	ns	-	-	ns	-
(77)	7-9 (8.2)	M/F	–	I(F)/D(M)	–	I	ns
(78)	8-9 (8.5)	M	-	ns	-	-	ns
(69)	6-15 (9.45)	M	ns	I	–	I	–

Whether the study focused on boys or on both boys and girls is specified in the ‘Sex’ column. I, increased; D, decreased; ns, no relationship.

Overall, out of seven studies that examined DHEA in relation to social communication impairments or overall symptom severity, five found no relationship with DHEA levels (69, 71–73, 75), all examining only boys with ASD compared to controls. Out of four studies that examined DHEAS, three found no relationship between DHEAS and social communication (73, 75, 77, 78), with only one of these studies including both girls and boys (77).

Considered together, these findings suggest increased levels of DHEA in ASD children and generally unaltered DHEAS levels, with differences in androgen-pathways in ASD children. Increased activity in androgenic enzymes like CYP17A1 and SULT2A1 may drive higher DHEA production. Most studies did not find significant associations between DHEA(S) levels and social or communication impairments in ASD or with symptom severity. Notably, the research predominantly focuses on boys, leaving a gap in understanding these patterns in girls with ASD.

### 3.3 Results for systematic review 3: impact of DHEA and DHEAS on repetitive behaviors and restricted interests

This section examines the potential links between altered adrenarche timing, variation in DHEA and DHEAS levels, and the presence of repetitive behaviors and restricted interests in ASD. By analyzing the relationship between adrenal hormones and these ASD traits across different ages, this section aims to clarify if hormonal variations during middle childhood contribute to shifts in these ASD-related phenotypes.

#### 3.3.1 Different sampling methods

A summary of each study reviewed for systematic review 3 can be found in **Table 7**. Studies differed in methods of assessing DHEA(S) and ASD traits. He et al. (72) measured only DHEA, two studies measured only DHEAS (73, 81), and three studies measured both (74, 75, 77). Three used the Autism Diagnostic Observation Schedule and the Autism Diagnostic Interview to measure ASD traits; Tordjman et al. (73) utilized the first edition, and two used the second edition (74, 75). In addition, Lakatosová et al. (75) used the Behavior Problems Inventory, He et al. (72) used the Repetitive Behavior Scale, and Erbay et al. (81) studied children with chronic tic disorder or Tourette’s syndrome via the Yale Global Tic Severity Scale. Two studies utilized the Childhood Autism Rating Scale (CARS), which includes measures related to repetitive behaviors and restricted interests, including object and body use and adaptation to change (71, 77), however Majewska et al. (77) only focused on overall CARS scores, not reporting any results for specific CARS measurements. Four studies assessed only boys (72–75), and two assessed both boys and girls (77, 81).

**Table 7:** Articles selected for review for systematic review 3 (Q3a-d; *impact of DHEA and DHEAS on repetitive behaviors and restricted interests*), alongside extracted information on participant sex and age, which measurements were used to assess adrenarche and ASD traits, and key findings in terms of DHEA and DHEAS.

Authors DOI (Type of study)	Title	Participants	Measurements Adrenarche ASD traits		Key findings
(71) Hassan et al. (2019) doi:10.1007/s12031-018-1225-9 (Cross-sectional)	Possible metabolic alterations among autistic male children: Clinical and biochemical approaches	146 boys aged 4 - 11, divided into 73 males with autism and 73 healthy age- and sex-matched children.	Serum concentrations of markers of mitochondrial dysfunction, oxidative stress, and heavy metals, including DHEA, cortisol, and free T, were determined.	Neuropsychological assessments, including CARS, short sensory profile, and intelligent quotients, were conducted.	Evidence of mitochondrial dysfunction, low cholesterol levels in ASD boys. Significantly lower total cholesterol, cortisol, and estradiol levels, as well as higher DHEA and free testosterone levels.
(72) He et al. (2023) doi:10.1186/s12888-023-04586-2 (Cross-sectional)	Analysis of salivary steroid hormones in boys with autism spectrum disorder	55 boys with ASD and 24 neurotypical controls.	Saliva samples were collected to obtain measures of cortisol, DHEA, and pregnenolone.	Utilized the child behavior checklist, autism behavior checklist, social responsiveness scale, and the repetitive behavior scale to assess ASD symptom severity.	Pregnenolone but not DHEA was associated with the RBS score, and boys with higher pregnenolone had a higher risk of ASD. Only pregnenolone was linked to autism behavior checklist scores.
(73) Tordjman et al. (1995) doi:10.1007/BF02179290 (Cross-sectional)	Plasma androgens in autism	39 autistic subjects (31 prepubertal, 8 postpubertal), 12 cognitively impaired subjects (all prepubertal), and 21 normal control subjects (10 prepubertal, 11 postpubertal).	Serum concentrations of DHEAS, testosterone (T), and whole blood serotonin (5-HT) were determined, and pubertal maturation was assessed via Tanner Stage criteria.	Behavioral assessments via the autism behavior checklist, autism diagnostic observation schedule, Vineland adaptive behavior scales, and a scale for lifetime aggression, which test for autism severity, self-injurious behaviour, and stereotypies.	No correlations with DHEAS and autism severity, self-injurious behavior, aggression, or stereotypies.
(74) Jansáková et al. (2020) doi:10.1038/s41398-020-01017-8 (Cross-sectional)	Alteration of the steroidogenesis in boys with autism spectrum disorders	62 prepubertal boys with ASD and 24 age- and sex-matched neurotypical controls.	Plasma samples were collected to obtain 82 biomarkers of steroidogenesis, including DHEA, DHEAS, progesterone.	Children were diagnosed using the DSM-IV criteria through the Autism Diagnostic Observation Schedule, 2nd revision (ADOS - 2), and the Autism Diagnostic Interview-Revised (ADI-R).	Correlations were found between behavioral indices and circulating steroids, with the best correlation found for social interaction. DHEA and DHEAS were both found to be strong predictors of ASD subdomains: social interaction, communication and language, and repetitive, restricted and stereotyped interests.
(75) Lakatosová et al. (2022) doi:10.30773/pi.2021.0094 (Cross-sectional)	The relationship of steroid hormones, genes related to testosterone metabolism and behavior in boys with autism in Slovakia	172 boys with a mean age of 5.2 years of age, and 135 neurotypical boys with a mean age of 11.	Plasma samples were obtained to determine the concentration of testosterone, estradiol, DHEA, DHEAS, and sex hormone binding globulin (SHBG).	Boys underwent behavioral testing via ADI-R and ADOS - 2 to evaluate the core symptom severities of ASD, the behavior problem inventory questionnaire assessed stereotyped behavior, aggression, self-injurious behaviors.	SHBG, DHEA, and DHEAS positively correlated with age. Negative relationship between SHBG levels and restricted, repetitive behaviors, and a positive relationship between testosterone levels and frequency of stereotyped behavior. SHBG levels and testosterone are related to the severities of restricted and repetitive behaviors in boys.
(77) Majewska et al. (2014) doi:10.1007/s00787-013-0472-0 (Cross-sectional)	Marked elevation of adrenal steroids, especially androgens, in saliva of prepubertal autistic children	78 prepubertal autistic male and female children from two age groups (3 – 4 and 7 – 9 years old) along with 70 neurotypical controls.	Salivary concentrations of 22 steroids, including androstenediol, DHEA, DHEAS, DHEA - 7o, pregnenolone, and androsterone, were determined.	Each child was rediagnosed by clinicians according to the DSM–IV criteria for ASD, and severity of traits was assessed according to the childhood autism rating scale (CARS).	More abnormal development (e.g. fine motor skills, speech delay, hypotonia, poor sleep) and hyperactivity was found in ASD children. In older ASD girls, CARS scores correlated positively with multiple steroids, including DHEA, DHEAS, DHEA - 7o, androstenediol, but older boys exhibited weak negative correlations with DHEA and DHEAS only. In younger ASD girls, CARS scores negatively correlated with androsterone, DHEA - 7o, and DHEA.
(81) Erbay et al. (2016) doi:10.1016/j.jocrd.2016.04.002 (Cross-sectional)	Testosterone and DHEA-S levels with chronic tic disorder in children	26 patients with chronic tic disorder or Tourette’s syndrome aged 7 – 12 who had not entered puberty and 25 healthy controls.	Serum levels of testosterone, DHEAS, and cortisol were measured.	Diagnoses were confirmed via DSM-IV criteria and the kiddie schedule for affective disorders and schizophrenia for school-age children.	T and DHEAS levels were higher in the patient group compared to the control group, but cortisol levels did not differ between the two groups. There were no significant correlations between androgen levels and tic severity.

#### 3.3.2 The impact of DHEA and DHEAS on repetitive behaviors and restricted interests

Most studies did not find any relationship between DHEA(S) and repetitive and restricted behaviors in children with ASD. While Lakatosová et al. (75) found a negative correlation between DHEAS and restricted and repetitive behaviors in boys aged 2-5, they did not find a significant relationship with DHEA or DHEAS in boys aged 5–6 or aged 7-9. Similarly, Tordjman et al. (73) and He et al. (72) found no relationship between DHEAS or DHEA, respectively, and repetitive behaviors in boys aged 3-8. Erbay et al. (81) found higher DHEAS levels in girls and boys aged 7–12 with chronic tic disorder or Tourette’s syndrome; however, there was no correlation with tic severity. In contrast, Jansáková et al. (74) found a significant relationship between both DHEA and DHEAS and restricted and repetitive behaviors in boys with ASD aged 4-6, and Majewska et al. (77) found a positive correlation between DHEA (but not DHEAS) and CARS scores autistic girls aged 7-9, and a negative correlation in boys aged 7-9. Hassan et al. (71) found no significant relationship between DHEA and CARS items in boys 4-11.

Most studies found no consistent relationship between DHEA or DHEAS levels and repetitive behaviors or restricted interests in ASD, though a few studies noted correlations within specific age and gender subgroups. Overall, the findings suggest limited and variable associations, highlighting a need for more research in this area.

## 4 Discussion

Autism has many causes, but they all converge on the presence of maladaptation in central aspects of social development. Given that middle childhood represents a key period during the development of social abilities in children, it should not be unexpected that alterations to this stage may modulate the onset, expression or symptoms of ASD or autism spectrum traits that develop and become evident between the ages of about 7 and 11. This hypothesis was motivated by the discovery by Zhang et al. (20) of a genetically-based subtype of ASD that is diagnosed mainly during middle childhood, and that involves the expression of a suite of psychological traits, such as increased internalizing, that are notably distinct from those in earlier-diagnosed forms.

The main goal of this article has been to present and evaluate the hypothesis that the social adaptations initiated by adrenarche, mediated by increased expression of DHEA and/or DHEAS, are selectively altered or underdeveloped in this later-developing subtype of autism. These social adaptations include, for example, the acquisition of sociocultural norms, local to global shifts in information processing, development of advanced theory of mind skills including recursion (‘he thinks that she thinks that…’), complex moral reasoning, enhanced social communication skills including prosody of speech, sarcasm, irony, verbal competition, and gossip, increasingly-complex social competition in play, and an increased sense of gender and sexual identity (15). Indeed, from an evolutionary perspective, humans exhibit an extraordinarily long pre-adult period, including a stage unique to humans and other great apes, middle childhood, when the brain is nearly fully developed in size while the body remains relatively small (16, 17). Diverse evidence indicates that the adaptive significance of middle childhood involves social learning, such that children in this stage have been selected to optimally (and in relatively safe contexts) acquire key social skills that will be beneficial in later life in the contexts of adult social and sexual competition and cooperation (15, 29). As such, middle childhood is expected to represent an important critical period in social development, making it a potential point of vulnerability with regard to underdeveloped or dysregulated social maturation, and with regard to an increase in social challenges that may make pre-existing autism-related traits more evident and problematic.

The primary goals of the systematic reviews conducted have been to evaluate the presence and strength of evidence relating adrenarche and DHEA and/or DHEAS to ASD and autism-related traits, in endocrinological, neurological, and psychological contexts. The main findings of the systematic reviews are two-fold. First, considerable evidence links both DHEA and DHEAS with a set of psychological and psychiatric traits related to ASD, especially in the context of internalizing and externalizing disorders. Findings in this regard point toward increased social anxiety (41, 43, 44, 48, 54), potentially mediated by the role of DHEA and DHEAS in responses to social stress (40). DHEA and cortisol become increasingly coupled throughout puberty (46, 58), potentially to facilitate adaptive stress responses in the context of increasing social and emotional demands (64, 65). Girls with internalizing symptoms and social anxiety display sharper increases in DHEA during social challenges compared to boys and adolescents without internalizing symptoms, and elevated cortisol/DHEA ratios were linked to more persistent depressive symptoms; increased negative life events and ruminative coping styles in these girls may result in a more chronic release of DHEA that contributes to maladaptive stress responses (40, 62). Elevated DHEA and DHEAS levels may also be related to higher levels of social anxiety through their associations with increased pituitary gland volume; larger pituitary gland volumes have been associated with HPA axis hyperactivity, potentiating heightened stress sensitivity that increases risk for psychopathology (48). Elevated DHEA and DHEAS levels in girls have thus been associated with a higher probability and increased severity of anxiety, particularly social anxiety (54), and higher symptom scores for aggression, ADHD, ODD, and OCD have been found in children with premature adrenarche (which is associated with higher DHEA) (41, 44). These findings are generally consilient with the middle childhood autism phenotype described by Zhang et al. (20), which is characterized by a trajectory of intensifying social-emotional difficulties during middle to late childhood; this later-developing ASD subtype also exhibits moderate-to-high genetic correlations with internalizing disorders, ADHD, and trauma-related factors (e.g. childhood maltreatment, adverse childhood experiences, and PTSD). Taken together, the studies reviewed here thus support the idea that alterations to aspects of DHEA and DHEAS metabolism in middle childhood may be involved in phenotypes associated with the later-diagnosed subtype of ASD.

The second line of evidence reviewed here implicates elevated DHEA levels in children with ASD, aligning with the results from a recent meta-analysis by Wang et al. (82) who found significantly higher levels of androgens, including DHEA, androstendione, and testosterone, along with weak or negative associations with DHEAS, among individuals diagnosed with ASD compared to controls. Altered levels of DHEA precursors and metabolites, as well as increased activity of enzymes responsible for DHEA synthesis and metabolism, including increased CYP17 17,20-lyase activity, may increase DHEA availability (74, 79, 80). Gasser et al. (70) found a low ratio of glucocorticoids to androgens in children with ASD, further supporting a role for increased CYP17A1 activity and a shift toward increased production of androgens. While the source of DHEA could be of neural or gonadal origin, studies assessing prepubertal individuals were able to rule out gonadal sources (74, 77), and DHEA levels exhibit strong positive association with levels of total testosterone and free testosterone, suggesting a common adrenal source (67). Hormonal imbalances involving increased DHEA and testosterone provide a potential biochemical framework that could underpin the neurobiological mechanisms linking DHEA and DHEAS to the development of autism-related social-emotional traits, especially in girls (41, 44, 49). Such findings are consistent with hypotheses that posit roles for elevated androgen levels in the development of ASD (22), and with effects of prenatal DHEA levels on aspects of infant emotional reactivity (83). Considered together, these primary findings support the hypothesis that alterations to DHEA, adrenarche, and middle childhood social adaptations may mediate the expression of cases of ASD with a middle to late childhood age of expression and diagnosis.

Although direct evidence linking DHEA and DHEAS to social-emotional behavior is limited, neuroimaging findings from the reviewed articles provide further insights into how these hormones may influence psychological and cognitive traits. Thus, for example, among boys and girls around 10 years in age, higher levels of DHEA levels have been associated with reduced affect-related brain activity in the mid-cingulate cortex (for both sexes), and, among girls only, in various cortical and subcortical regions (42). Higher DHEA levels have also been associated with increased externalizing symptoms in females, an association that was partly mediated by posterior insula activation to happy facial expressions. Further implicating effects on emotionality, DHEA has been found to influence amygdala-IFG connectivity, which is involved in downregulating amygdala responses to emotional stimuli, with elevated levels relating to increased connectivity and anxiety in boys but reduced connectivity and anxiety in girls (52). Sex differences in the effects of DHEA and DHEAS on amygdala connectivity may stem from increased androgen receptor density in boys due to elevated prenatal testosterone exposure, where lower testosterone levels and fewer androgen receptors in girls could allow DHEA and DHEAS greater binding opportunities (52).

Barendse et al. (43) found that elevated DHEA in boys increased amygdala-visual cortex connectivity, contributing to heightened social anxiety, while elevated DHEAS in girls decreased amygdala connectivity to the left insula and FFA, resulting in reduced social anxiety (43). Reduced amygdala-insula connectivity may diminish the intensity of emotional responses to negative stimuli, while reduced connectivity with the FFA may weaken the recognition and interpretation of emotional facial expressions, potentially reducing attention to socially threatening cues by dampening emotional reactivity but also limiting the ability to detect and appropriately respond to nuanced social and emotional signals (84, 85). Increased amygdala-visual cortex connectivity in boys may also heighten sensitivity to social cues by amplifying the amygdala’s response to novel or ambiguous social stimuli, leading to an over-emphasis on these cues in social situations (86). In this context, individuals with ASD often exhibit impaired judgment of facial expressions, inaccurately labeling neutral faces as negatively valenced, with reduced eye contact correlating with increased amygdala reactivity and threat ratings (87). Amygdala dysfunction is also common in ASD and has been associated with social anxiety, sensory over-responsivity, and social-communication impairments (88). Together, the evidence suggests that elevated DHEA and DHEAS can influence cognitive and emotional behaviors by altering brain activation and functional connectivity in ways that can promote either adaptive or maladaptive development.

Further neuroimaging studies support the role of DHEA and DHEAS in mediating middle childhood adaptations. Heightened DHEA levels have thus been associated with negative structural covariance between the amygdala and the occipital lobe, parietal lobe, and ACC (cortical thickening in these regions decreased with increasing amygdala volume), resulting in improved visual awareness, visuo-motor dexterity, and reduced attention problems, respectively; DHEA may thus promote a shift from a bottom-up, amygdala-driven attentional processing system, strongly influenced by emotional responses, to a top-down, more deliberate and goal-directed system (89). Attentional control develops significantly in middle childhood as children improve their ability to suppress distractions and shift attention, potentially to allow for better sustained attention; initially strong distractibility in children aged 6–10 decreases over time, indicating rapid learning and adaptation during this period (90). Furthermore, a positive age-specific association between DHEA and cortical thickening was found in the right temporoparietal junction (rTPJ) between the ages 7-12 (91). The rTPJ is involved in directing attention towards relevant social stimuli, guided by the ACC to potentially help prioritize and attend to relevant social cues (92). More complex ToM abilities arise starting around age seven, including understanding recursive mental states (understanding that someone can have a belief about another person’s belief), nonliteral language (including irony and sarcasm), and recognizing faux pas, contributing to successful social integration (18). The rTPJ’s role within the ventral attention and ToM networks enables it to update one’s understanding of another’s mental state in response to new information, thus facilitating the development of cognitive empathy, which develops between the ages of around 10-12 (93). Overall, DHEA and DHEAS may thus facilitate the acquisition of emotional processing and regulation abilities, attentional control, and enhanced ToM development during middle childhood.

**Figure 1:**
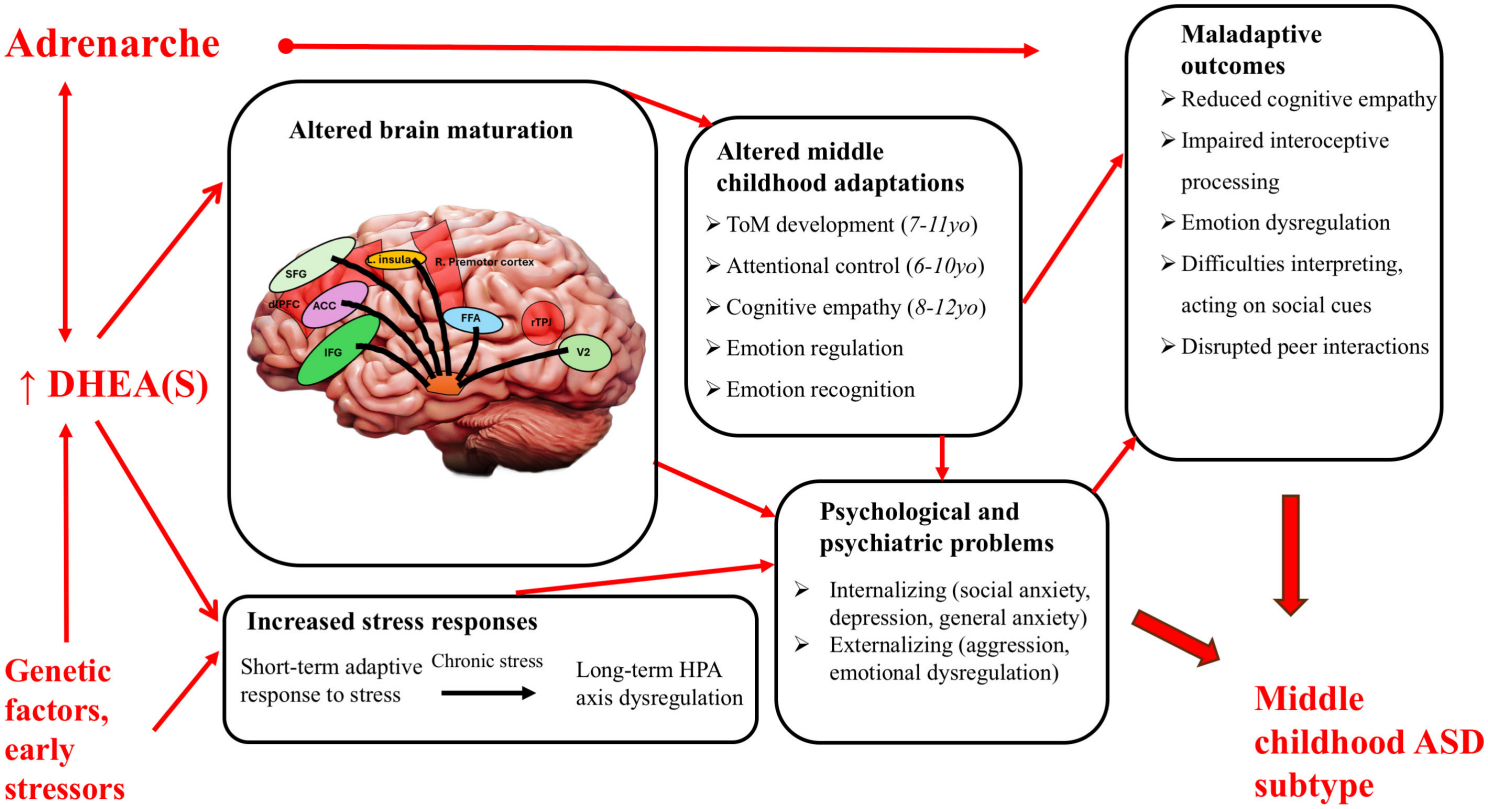
Simplified theoretical model for role of DHEA(S) in the development of middle childhood ASD subtypes. See text for details.

Taken together, these findings suggest that high levels of DHEA and DHEAS, developing through early adrenarche timing and elevated social stress reactivity, may impact the acquisition of middle childhood adaptations, leading to maladaptation in the context of autism-related traits (**Figure 1**). By this model, alterations to levels of DHEA and DHEAS impact the adaptive function of these hormones with regard to social opportunities and threats. While moderate DHEA levels can thus promote positive adaptations, including limiting emotional salience in stressful social contexts, potentially to support peer integration and bottom-up to top-down attentional processing (89), exaggerated levels due to early adrenarche timing or other causes may lead to maladaptive outcomes, including an increased sensitivity to threatening and ambiguous social stimuli and reduced interoceptive processing (42, 43). These results are consistent with the middle childhood autism trajectory described by Zhang et al. (20), which is characterized by positive genetic correlations with internalizing disorders and social-emotional difficulties that escalate during this developmental stage. By positioning adrenarche as a key biological substrate for these changes, this study underscores the potential for middle childhood to act as a critical period in shaping ASD traits through the interplay of endocrine, environmental, and neurodevelopmental factors. Such insights reinforce the need to further investigate the mechanisms through which altered adrenarche timing and DHEA(S) levels contribute to the heterogeneity of ASD, with a focus on middle childhood as a sensitive window for intervention and support. Such investigations should include prospective longitudinal studies of both DHEA(S) hormone levels and social behavior, in relation to the nature and intensity of social challenges, across the adrenarche transition.

One important limitation of this study is the variability in methodologies and sample characteristics across the reviewed articles, particularly the limited number of studies directly assessing how DHEA and DHEAS impact traits associated with ASD, especially repetitive behaviors and restricted interests (RRBs). Results from the reviewed studies underscore this gap, as there were few studies of relationships between DHEA and DHEAS levels and these traits. In contrast to these results, DHEA, or DHEAS levels have been reported as higher among individuals with childhood Obsessive Compulsive Disorder (OCD) (94), and Tic disorder (95) compared to controls, and for both disorders the usual age of onset occurs around the age of adrenarche, for pediatric cases. Given the comorbidities of ASD with OCD and Tic disorder (e.g., (96)), and similarities of OCD and Tic-related behaviors to some autism-related RRBs, these findings suggest the possibility that RRBs among individuals with the later-onset subtype of ASD may, like some other ASD-related traits, differ in their manifestations and causes from individuals with early-onset ASD.

A second limitation identified here is a substantial underrepresentation of girls in studies examining DHEA and DHEAS levels and their effects on ASD traits, leaving critical gaps in understanding potential sex-specific patterns. Considering the developmental trajectories of ASD traits and the dynamic changes in DHEA and DHEAS during middle childhood, future research should prioritize longitudinal designs to better capture how hormonal fluctuations may influence social and cognitive adaptations over time. These studies should include diverse populations to address gaps in gender and ethnic representation and ensure findings are broadly applicable. Indeed, only one of the populations represented in this review, from China (72), was not ethnically Caucasian or Middle Eastern. Investigations should also aim to elucidate the neurobiological mechanisms underlying these relationships, particularly the roles of DHEA and DHEAS in modulating neural circuits involved in social cognition, including the amygdala, insula, and prefrontal cortex, to provide deeper insights into how hormonal dysregulation during critical developmental windows contributes to the heterogeneity of ASD phenotypes.

## Data Availability

The original contributions presented in the study are included in the article/**Supplementary Material**. Further inquiries can be directed to the corresponding author.
