# Supplementary material for: Adrenarche, social cognition, and the development and evolution of autism spectrum traits
Source: Front Psychiatry. 2025 Sep 9;16:1576392. doi: 10.3389/fpsyt.2025.1576392 (PMC12455462; doi:10.3389/fpsyt.2025.1576392)
Supplement: Supplementary file 1 [file SupplementaryFile1.docx]

**Supplementary Figure 1.** PRISMA flow diagram for hypothesis 2.1a

**
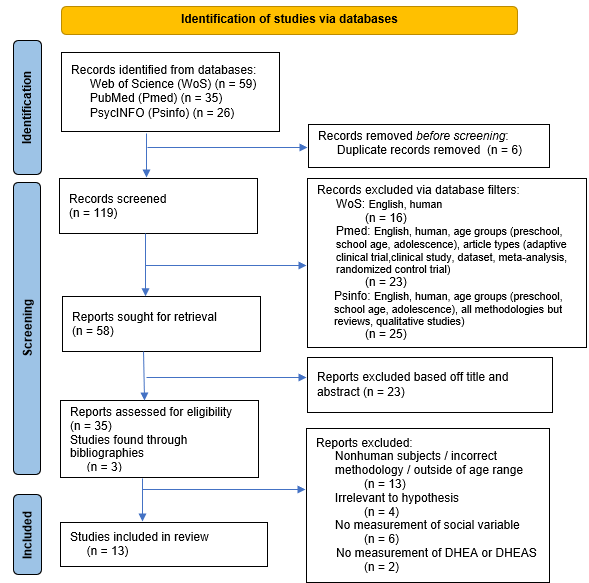
**

**Supplementary Figure 2.** PRISMA flow diagram for hypothesis 2.1b

**
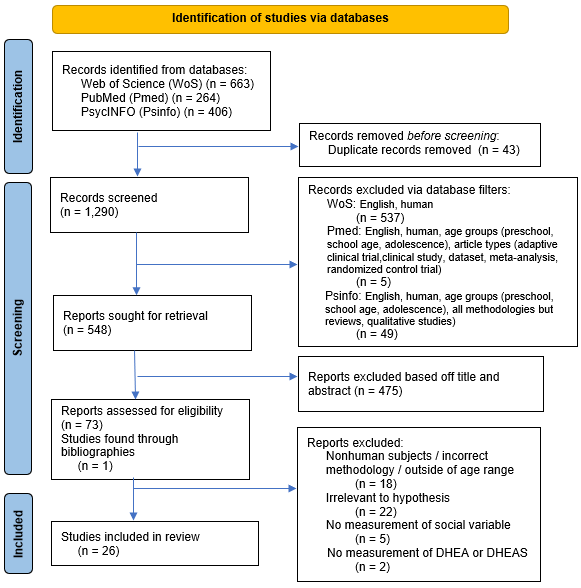
**

**Supplementary Figure 3.** PRISMA flow diagram for hypothesis 2.2a


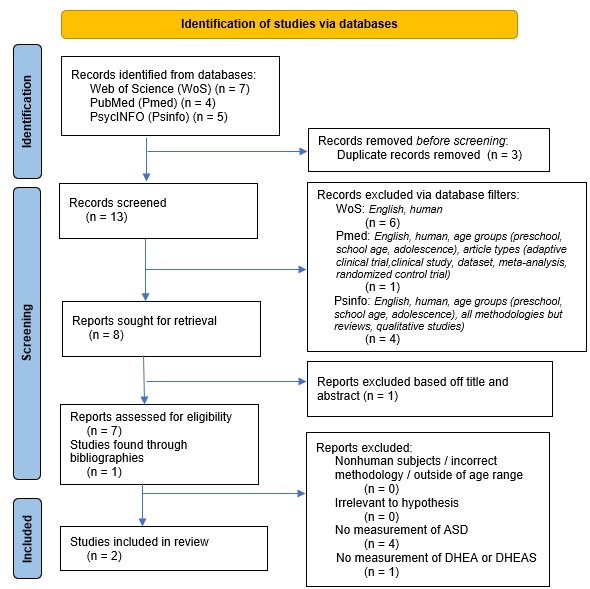


**Supplementary Figure 4.** PRISMA flow diagram for hypothesis 2.2b


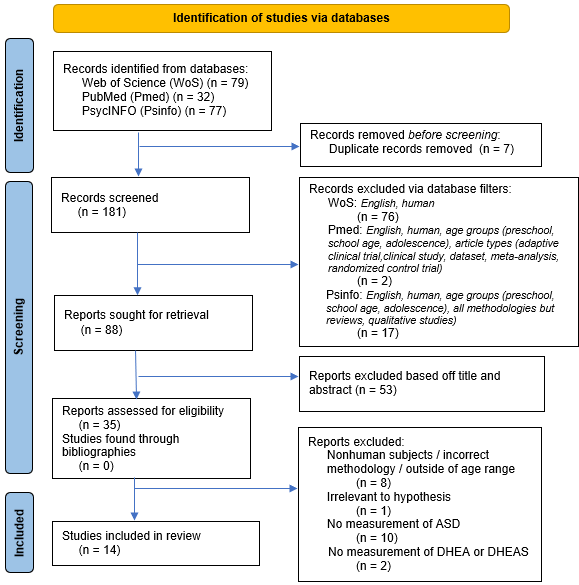


**Supplementary Figure 5.** PRISMA flow diagram for hypothesis 2.3a


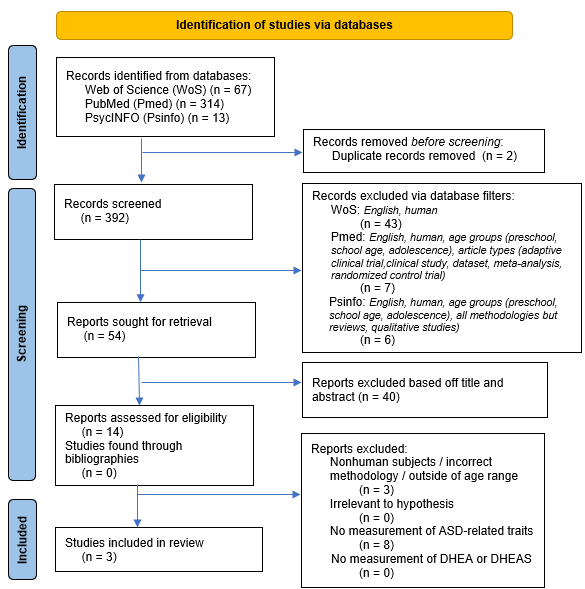


**Supplementary Figure 6.** PRISMA flow diagram for hypothesis 2.3b


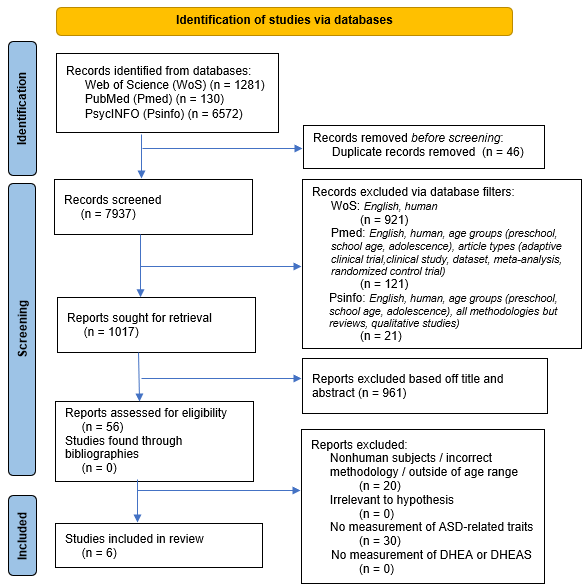


**Supplementary Figure 7.** PRISMA flow diagram for hypothesis 2.3c


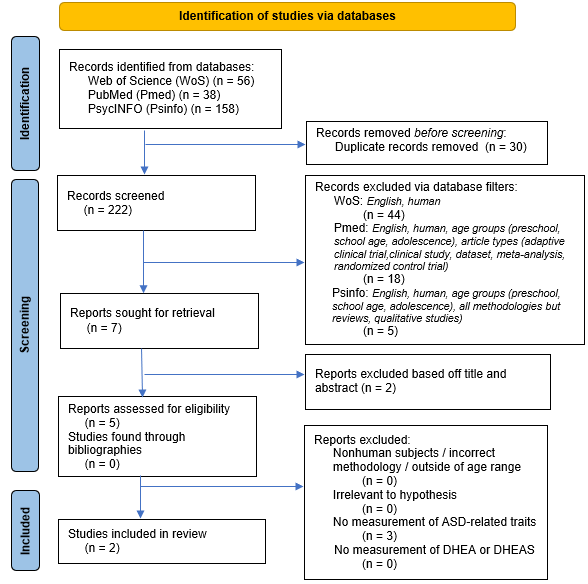


**Supplementary Figure 8.** PRISMA flow diagram for hypothesis 2.3d

**
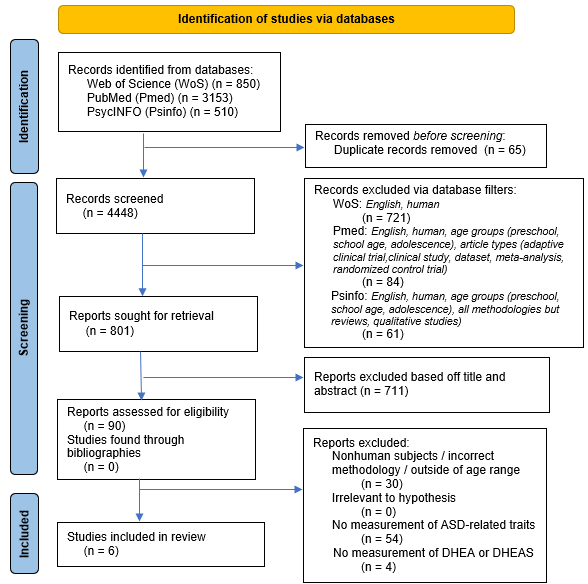
**
